# Skeletal Lesions in Human Tuberculosis May Sometimes Heal: An Aid to Palaeopathological Diagnoses

**DOI:** 10.1371/journal.pone.0062798

**Published:** 2013-04-24

**Authors:** Kara L. Holloway, Karl Link, Frank Rühli, Maciej Henneberg

**Affiliations:** 1 Biological Anthropology and Comparative Anatomy Research Unit, The University of Adelaide, South Australia, Australia; 2 Centre for Evolutionary Medicine, Institute of Anatomy, University of Zürich, Zürich, Switzerland; Institut de Pharmacologie et de Biologie Structurale, France

## Abstract

In three to five percent of active cases of tuberculosis, skeletal lesions develop. Typically, these occur on the vertebrae and are destructive in nature. In this paper, we examined cases of skeletal tuberculosis from a skeletal collection (Galler Collection) with focus on the manifestation of bony changes due to tuberculosis in various body regions in association with antibiotic introduction. This skeletal collection was created in 1925–1977 by a pathologist at the University Hospital in Zürich, Ernst Galler. It includes the remains of 2426 individuals with documented clinical histories as well as autopsies. It contained 29 cases of skeletal tuberculosis lesions. We observed natural healing of vertebral lesions through several processes including fusion of vertebrae, bone deposition and fusion of posterior elements. In these cases, we observed a higher frequency and proportion of bone deposition and fusion of posterior vertebral elements where pharmacological agents were used. There were also four cases of artificial healing through surgically induced posterior spinal fusion. With the introduction of pharmaceutical treatments, the number of individuals with multiple tuberculous foci decreased from 80% to 25% when compared to individuals who did not receive any drug therapy. Investigation of comorbidities showed that pneumonia, pleuritis and being underweight were consistently present, even with pharmaceutical treatment. Our results have applications in palaeopathological diagnoses where healing and consequent bone deposition may complicate differential diagnoses.

## Introduction

In palaeopathology, diagnosis of tuberculosis is rare (review in [1]) because skeletal lesions occur in no more than 5% of all active cases while these lesions are not always pathognomonic. Therefore it is important to present well documented cases of skeletal TB involvement to improve the possibility of palaeopathological diagnosis.

Tuberculosis (TB) is primarily a pulmonary disease that can affect individuals of all ages, but occurs mostly among individuals with lowered immune function [2], [3]. The most common signs and symptoms of pulmonary TB include localized damage to the lungs resulting in respiratory distress (coughing, difficult breathing, bloody sputum and chest pain), as well as a general “wasting” that may include fatigue, weight loss and pallor [4], [5]. The clinical manifestation of TB varies depending on the immune status of an individual after becoming infected by the causative agent, *Mycobacterium tuberculosis* [6]. Signs and symptoms can range from an active, debilitating illness in those with low immunity to a chronic, sub-clinical, latent infection in those with sufficient immunity to control the bacterium [7], [8].

In three to five percent of cases of active TB, osteolytic skeletal lesions develop; these occur mainly on the vertebrae [9], [10]. The typical bone lesion for TB is destruction of the anterior region of vertebral bodies with subsequent collapse of the spine [10], [11]. However, posterior regions are affected in some cases [7]. Usually only up to four vertebrae are affected in this process and fusion of partly destroyed vertebrae is commonly observed in the later stages of the disease. Other diseases that can produce similar spinal lesions include brucellosis, fungal infections, pyogenic osteomyelitis, vertebral fractures and neoplasms [10], [12], [13]. A careful differential diagnosis can eliminate other diseases as potential causative agents. Bone lesions caused by TB can occur in other locations on the skeleton, but occur most frequently at major joints such as the hip and knee. These sites account for 15% to 30% and 10% to 20% of non-spinal cases of skeletal TB, respectively [10], [14]. The typical lesion of these sites is destruction of the articular surfaces of the joint [10], [11]. No new bone formation is expected; except where the disease has been inactive for a long time [6]. In general, palaeopathological diagnoses based on skeletal lesions do not include TB when bone deposition has occurred as this is not considered characteristic of the disease [11]. There is, however, a possibility of bone deposition occurring in TB cases. This needs to be investigated on bone specimens coming from patients with well-diagnosed TB.

Before the 1940's and 50's the only treatments available for TB were surgical interventions as well as a general improvement in an individual's immunity through rest, good nutrition and hygiene [6], [14], [15]. These treatments were provided at sanatoria, which increased in popularity in most countries from around the year 1854 [16].

Besides the fairly common rib resection procedure, surgical intervention included posterior spinal fusion which was considered necessary when extensive vertebral destruction had occurred. This procedure was developed in the United States by Dr Fred H. Albee in 1909 [17]. In his original article, Albee highlights the importance of immobilizing the intervertebral joints in order to allow ankylosis and consequently healing of the spine to occur. However, because the vertebral joints are always moving due to breathing and other normal activities, the use of braces and casts does not provide sufficient immobilization of the spine. Albee's original procedure involved using a bone graft from the individual's own tibia, which has been shown to help initiate bone deposition and ankylosis of the spinal joints. It was noted that not only did the grafts internally immobilize the spine, but also provided mechanical support, especially where the spine had collapsed due to severe destruction of vertebral bodies. The bone taken from the tibia also had vascularising ability; healing of the spine through formation of new blood vessels and return of blood flow to areas of the vertebrae severely damaged by TB were both observed.

In 1943, the first antibiotic to specifically combat TB, streptomycin, was produced, but did not become widely available until 1946 [18], [19]. In 1952, a second pharmacological agent called isoniazid (INH) was implemented into TB treatment regimes [20]. Para-aminosalicylic acid (PAS) was available earlier than this but was not as effective as INH. Unfortunately, these pharmacological treatments were unable to cure all individuals with TB but were certainly able to prolong the life of a person diagnosed with the disease. With the combination of immune based therapy and pharmacological intervention, mortality from TB declined from the 1800's until recently, when the development of drug resistance, the beginning of the HIV epidemic and a shift away from government funded medical support reversed the trend [21]–[23].

In our previous work, we have shown using historical records from both the Stadtarchiv (“city-archive”) and Staatsarchiv (“canton-archive”) in the city of Zürich (Switzerland) that TB was common there during the 19^th^ and early 20^th^ centuries (1893 to 1933). Data were obtained for both the city of Zürich and the entire Canton of Zürich (Switzerland comprises a number of smaller regions known as “cantons”). A comparison of TB mortality rates for The Netherlands, Denmark, Belgium and England and Wales was conducted, showing that at the beginning of the 20^th^ century TB mortality rates were between 150 and 200 per 100,000 living for all countries as well as the Canton of Zürich. However, in the city of Zürich, the mortality rate was higher; at 400 per 100,000. Many countries including Switzerland, show two peaks of TB mortality through time, the first occurring just before 1920 and the other between 1940 and 1950. These correspond to the first and second World Wars as well as the influenza outbreak in 1918 (Spanish flu) [24], [25]. During these periods, immunity of populations decreased because of food shortages, hard working conditions, overcrowding and military service [26]. Despite these peaks in mortality, a general trend of decreasing mortality from TB continued through time in Europe and North America.

In this study, our aim was to describe the skeletal manifestation of TB in the well-documented pathology collection covering the period from the early 20^th^ century to 1970s (Galler collection, Switzerland) in order to obtain more information about the processes of skeletal lesion formation and healing through time. This period covers the time where no effective pharmacological treatments for TB were available as well as the time of their introduction (late 1940s) and eventual widespread use. We investigated the number of regions of the body affected by the disease as well as co-morbidities in addition to TB.

## Materials and Methods

### Skeletal Collection

The skeletal collection used in this study is the Galler collection; initiated in the early 1900's by Ernst Galler and now stored at the Natural History Museum in Basel (Galler 1) and at the University of Zürich (Galler 2). The aim of Ernst Galler was to collect examples of skeletal lesions useful in studies of pathologies affecting bones. It contains the skeletal remains of 597 individuals (Galler 1) and 1829 individuals (Galler 2) from the Zürich Canton who died throughout the 20^th^ century. This collection is loaned to the University of Zürich from the Institute of Pathology. Galler 1 was sent to the Natural History Museum in Basel because of insufficient space to store this part of the Galler collection. We received permission to examine these materials from the University of Zürich, under specific guidelines of confidentiality. These individuals were from all backgrounds including, among others, construction workers, health workers and housewives. Medical records, autopsy reports, medical photographs and X-rays are available for many individuals in the collection and it is thus a useful sample for investigating the development of bone lesions with background knowledge of the soft tissue pathology. A summary of Galler 1 can be found in [27], stating that most specimens have original autopsy reports available, in addition to information on age, sex, origin and profession. The partially handwritten reports are approximately four pages in length each with detailed macroscopic and microscopic descriptions as well as a summary of clinical history, autopsy findings and medical treatment history. Descriptions of soft tissue pathology are given, however, for this study we were more interested in skeletal pathologies which were also described. There are available photographs of skeletal lesions taken at the time the specimens were deposited. Thus these photographs are of the quality obtainable with earlier photographic techniques, yet they demonstrate sufficently well skeletal lesions. There is no publication describing Galler Collection 2 at this time. It comes from somewhat later time period, has better quality autopsy reports and the specimens were available for direct study and digital photography.

Medical records and autopsy reports for individuals from the Galler Collection were consulted in order to compare changes in TB manifestation during 1925–1977 in Switzerland. For this study, we searched the Galler Collection database for individuals who had been diagnosed with any type of TB (pulmonary, skeletal, major joints, lymph nodes, etc.) during their life. The cause of death did not have to be TB. We found 69 individuals who met this criterion, however, only 29 of these had bone lesions possibly associated with TB. The remainder (40 individuals) were kept in the collection because they were good examples of other bone pathologies, although the individual may have had TB during their lifetime that did not result in skeletal lesions. All were autopsied during the 20^th^ century, but only one (Autopsy Number: 2289, Autopsy Year: 1968) died of TB. We investigated the possibility of other diseases causing the observed bone lesions and firstly consulted the medical records for any evidence of other causes. We reviewed the medical records to determine any evidence of TB at the site of a lesion. Where significant evidence was documented, we considered the lesion to be tuberculous in origin. Due to time and financial restrictions, radiography was not performed, but may be done in future studies. Differential diagnoses were also attempted based solely on skeletal lesions observed and several other possible diseases considered with focus on compression fractures, Paget's disease, osteomyelitis and neoplasms as these were considered the most probable other causes of spinal lesions in this sample (age range was 16 to 98 years). Septic arthritis was considered as a potential alternative diagnosis for cases involving joints such as the hip or knee. The criteria we used for macroscopic diagnosis of spinal TB were based on the guidelines described by [10] and [11]. These criteria included (at least two):

- Destructive lesions on the anterior region of the vertebral body; posterior regions of vertebrae remained unaffected.
- Usually 2–4 adjacent vertebrae were affected.
- Presence of kyphosis and/or scoliosis.
- The lower thoracic and upper lumbar vertebrae were affected (instead of the cervical or sacral regions).
- Intervertebral disc destruction.

We did not include minimal bone deposition as a criterion in this study because our aim was to show that bone deposition does indeed occur in TB. Since TB was common in Switzerland at this time, these cases likely represent common manifestations rather than unusual cases.

Individuals were grouped into three time periods based on the availability of pharmacological intervention in Switzerland, starting with streptomycin in 1946 [19]. The time periods are defined in this study as: before 1946 (first period), 1946–1950 (second period) and after 1950 (third period). The third period defines the time period where pharmacological agents (including antibiotics) became widely used to treat individuals. The second time period is used to ensure separation of the periods when antibiotics were not regularly used and when they were available to most individuals. Individual records used did not contain information about pharmacological treatment, but it can be safely assumed that medical practitioners in Switzerland would not deny an individual with TB treatment that was routinely available at the time. Indeed this is true; a Law was passed in 1928 in Switzerland, making it mandatory for cases of TB to be reported and treated [28]. Since all of the individuals in our study were autopsied after 1928, it can be expected that all were recorded and treated in some way.

**Figure 1:**
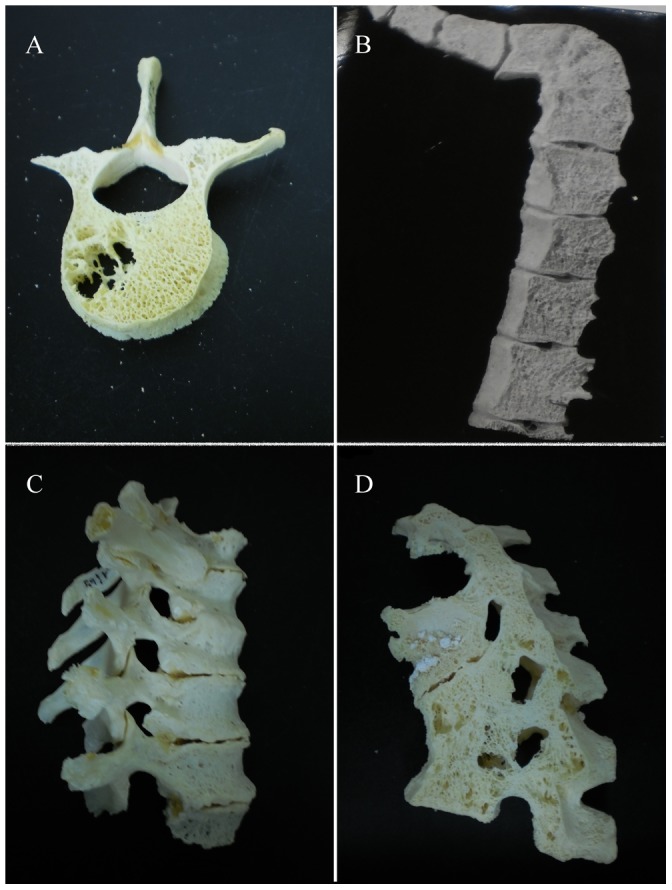
Examples of categories of spinal lesions due to tuberculosis used in this study. All cases presented here are from the Galler Collection and are fully described in Appendix S1. A) Autopsy Number: 1645, Autopsy Year: 1957, No evidence of “healing.” B) Autopsy Number: 411, Autopsy Year: 1955, fusion of vertebrae has occurred. C) Autopsy Number: 2461, Autopsy Year: 1969, bone has been deposited on the anterior bodies of affected vertebrae. D) Autopsy Number: 785, Autopsy Year: 1963, posterior elements (as well as vertebral bodies) have fused. Scale bar represents 10 mm.

**Figure 2:**
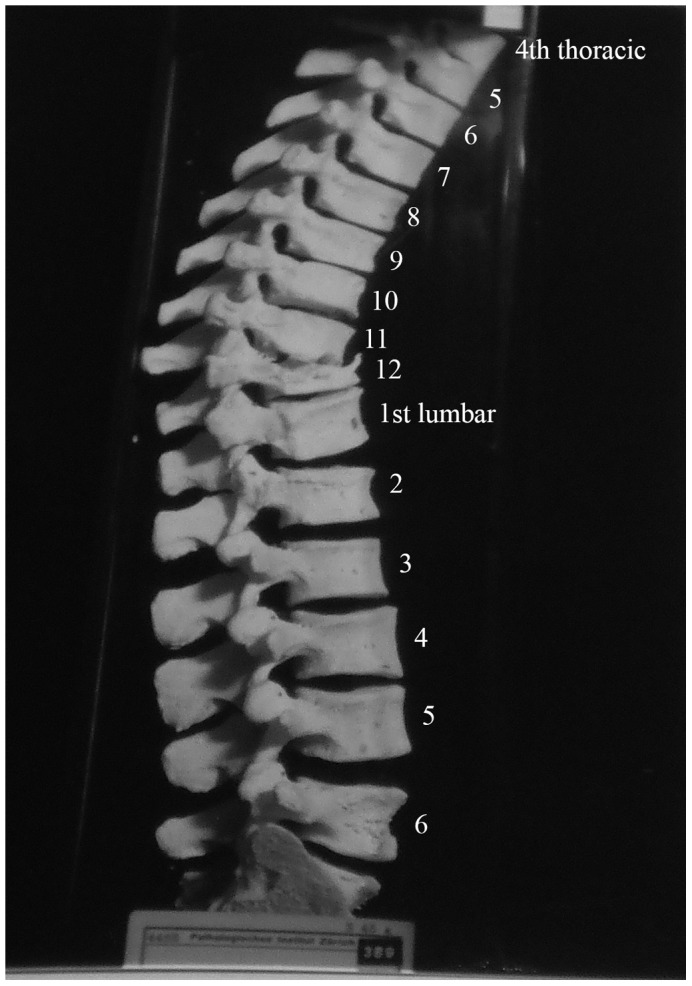
Autopsy Number: 751, Autopsy Year: 1939, Age: 35, Sex: Male.

Osteological lesions of the spine were examined and the level of “healing” was recorded. Healing is defined here as a cessation of osteolytic processes and evidence of healing processes such as ossification of ligaments and fusion of destroyed bones. We considered both spinal and non-spinal cases of TB in the analysis of skeletal lesion healing. Since this sample included a large number of spinal cases, we identified four types of lesions (Figure 1); no healing (A), fusion of vertebral bodies (B), bone deposition (C) and fusion of posterior elements (e.g. spinous processes) (D). These categories were created in order to separate different processes that occur during lesion healing in TB. Fusion of the anterior regions of vertebral bodies (B) is quite common in TB and is thus considered separately from bone deposition (C) and fusion of posterior elements (D); that do not occur very often in TB. (C) refers to bone deposited on any part of the vertebra, that does not result in fusion. It is uncommon for bone deposition to occur in TB [11] except through direct fusion of vertebrae. The fusion of posterior elements is rare and not recorded in the literature and thus we considered it separately from normal vertebral fusion and bone deposition, which are far more likely to occur. We considered fusion of posterior elements a sign of well healed TB, as this could not occur unless the disease had become inactive so that destructive processes usually occurring during active disease have stopped. We used these categories also in the cases of non-spinal TB. For example, in a case of hip TB, there can be no healing (Figure 2A), fusion of the joint (2B) and bone deposition (2C) as the lesion heals.

Information was obtained from medical records, autopsy reports and skeletal samples. In all cases there was agreement between the written reports and skeletal samples, but occasionally the records described lesions on bones of the skeleton that were not available for examination. Diagnoses were made by several individuals; firstly the original medical staff to treat these patients, secondly Dr. Karl Link through searching the database for the Galler Collection (this found any cases of TB) and finally through macroscopic investigations completed by Kara Holloway and Maciej Henneberg. What follows are case descriptions each illustrated by at least one photograph. Out of necessity, these photographs are of differing quality, depending on when they were taken (some in the early 20^th^ century). We also had to curtail the number of photographs due to space limitations. Thus, case descriptions should be treated as primary source of information while photographs only illustrate some major points.

Four individuals had received a posterior spinal fusion operation as a treatment for spinal TB. One was from the first period, one from the second and two from the third.

The number of organs affected by TB in individuals from the Galler Collection was also recorded by using information from medical records. Cases were divided into two groups: those with TB of a single region in the body and those with TB of more than one region. Co-morbidities were also recorded to provide further background information. Average ages at death were calculated along with standard error ( = standard deviation/√N). Table 1 shows an overview of the sample and the analysis groups.

**Table 1:** Overview of the cases of tuberculosis from the Galler Collection (Switzerland).

Analysis conducted	Number of individuals	Average Age (years)	Sex (M/F/?)
Full Collection	69	62±2	27/34/8
First time period (before 1946)	5	39±10	3/2/0
Second time period (1946–1950)	8	57±5	3/4/1
Third time period (After 1950)	51	65±3	21/28/2
Non-spinal TB	3	30±7	3/0/0
Analysis of bone lesion healing	25	60±4	11/14/0
Number of tuberculous foci	61	63±2	24/34/3
Co-morbidities	As above	As above	As above

## Results

### Cases of spinal tuberculosis

A total of 29 (42%) out of 69 individuals with medically diagnosed TB had observable skeletal lesions. This high frequency of skeletal involvement is unsurprising because the cases were specifically chosen for the presence of exemplary skeletal lesions. Of the spinal lesions observed, three (10%) were from the first time period, four (14%) from second time period and nineteen (66%) from third time period. Three additional non-spinal cases were discovered for the first (2 cases, 7%) and second (2 cases, 3%) time periods during the original search.

This first part of this study focused on spinal lesions as they are the most commonly recognized diagnostic lesion for TB. Ten representative cases are outlined below that show an array of pathological bone changes observed in the Galler Collection. All additional cases can be found in Appendix S1. Each case is supported by a photograph (Figures S1, S2, S3, S4, S5, S6, S7, S8, S9, S10, S11, S12, S13 and S14). The second part of the study describes the three cases of TB affecting a region of the skeleton other than the spine.

#### First time period (before 1946)

Description: Figure 2 shows the fourth thoracic to the six lumbar vertebrae of a 35 year old male. Spondylitis has affected multiple sites on the spine, including the anterior side of the eighth to twelfth thoracic vertebrae. The individual had back pain in 1937 (two years before death) and the spondylitis was diagnosed at this time. The anterior regions of thoracic vertebral bodies nine to twelve have bone erosion and thoracic vertebra twelve is almost completely destroyed. Lumbar vertebrae one and two are also affected by the disease process. Thoracic vertebra twelve and lumbar vertebra one have collapsed, causing an angulation of the spine. This has also occurred around the eighth and ninth thoracic vertebrae. The vertebral discs beginning at the level of thoracic vertebra eleven and ending at the first sacral vertebra are all destroyed. There is a small, circular lesion observable on the anterior surface of lumbar vertebra one (visible in Figure 2). It measures approximately 2 mm×4 mm. There is also a small amount of bone deposition on the anterior surface of the first sacral vertebra (visible in Figure 2). There is no evidence of healing of the spinal lesions.

In differential diagnosis, compression fractures, Paget's disease, osteomyelitis and neoplasms were considered, however, there were no mentions of these diseases in the medical records for this individual. This case shows anterior destruction of a single vertebra, which is consistent with TB. There is a mild kyphosis and the posterior elements are unaffected. In addition to the diagnosis of spondylitis TB, we considered this to be a case of TB.

**Figure 3:**
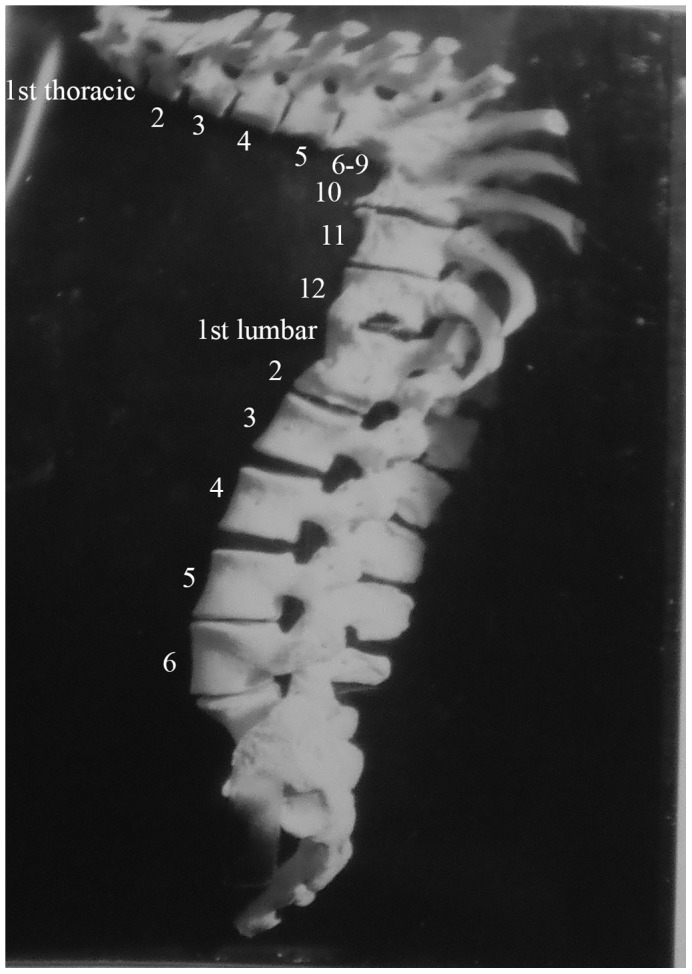
Autopsy Number: 793, Autopsy Year: 1936, Age: 78, Sex: Female.

Description: Figure 3 shows first thoracic to sixth lumbar vertebrae of a 78 year old female. She had spondylitis of the eleventh and twelfth thoracic as well as the first lumbar vertebrae. An abscess is present on the left side of the first lumbar vertebra. There is also healed spondylitis of thoracic vertebrae six through nine, involving fusion and kyphosis. The fusion is extensive and involves the entire vertebral disc surface of all vertebrae. There is an abscess on thoracic vertebra eleven, measuring approximately 4.5 mm×7 mm. The disc between thoracic vertebrae eight and nine has been destroyed. Other than thoracic vertebrae six to nine, eleven, twelve and lumbar one, all other vertebrae appear normal. There is reduction of intervertebral disc space between the fourth and fifth lumbar vertebrae as well as between the fifth and sixth lumbar vertebrae. This individual also had her right leg and a finger amputated due to TB. Healing has occurred in this case through fusion of vertebrae.

Differential diagnosis included compression fractures, Paget's disease, osteomyelitis and neoplasms, but these were not mentioned in the medical diagnoses. Additionally, in this case, more than four vertebrae were affected, but in two different regions of the spine. This was a result of two separate events. Considered separately, only four and three vertebrae are affected in these two separate instances. The skeletal lesions are destructive and occur on the anterior regions of vertebral bodies. In the upper thoracic region, the vertebral destruction has led to kyphosis. Posterior regions of the vertebrae were unaffected. Thus we considered this a case of TB.

#### Second time period (1946–1950)

**Figure 4:**
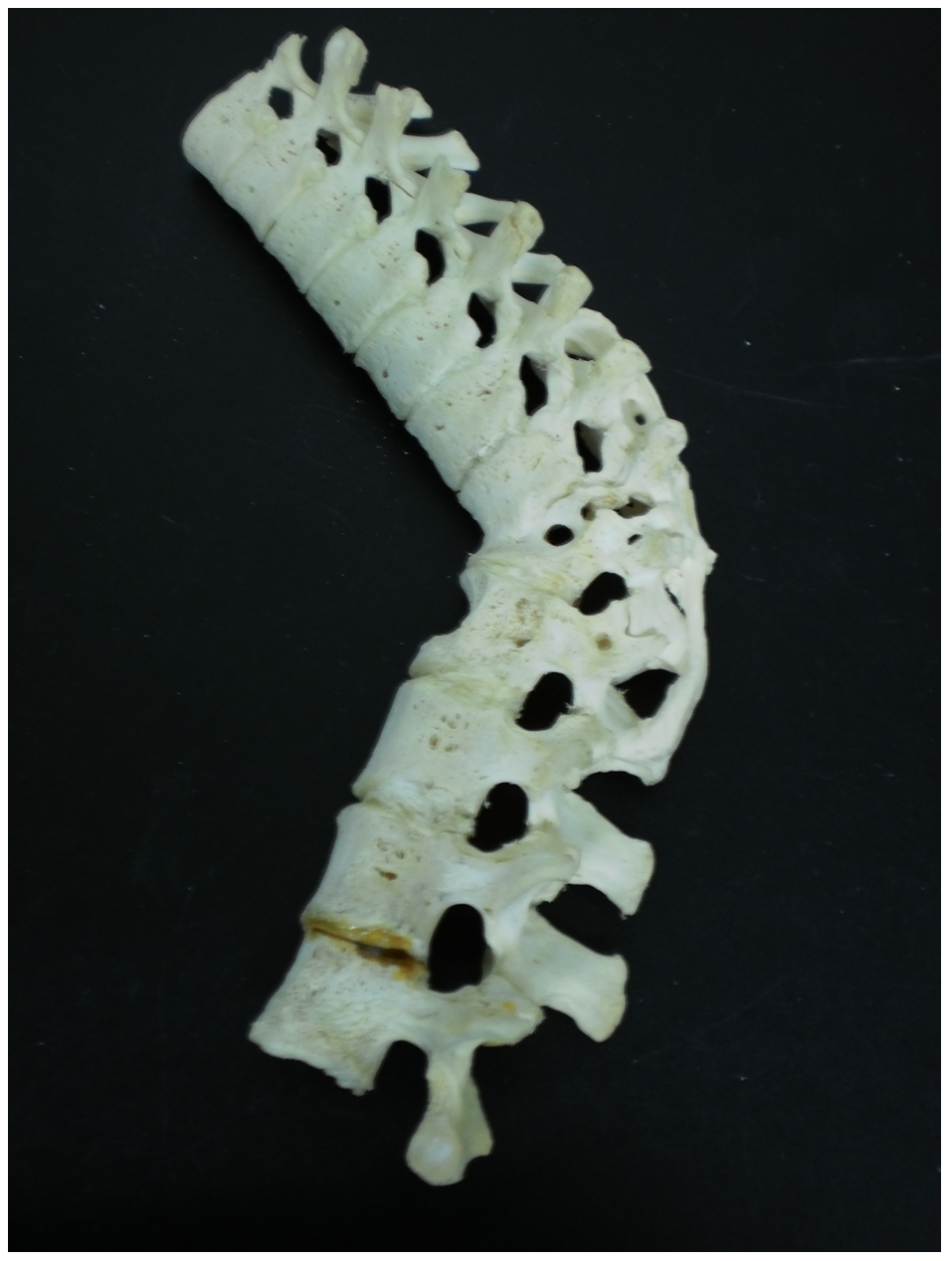
Autopsy Number: 732, Autopsy Year: 1949, Age: 65, Sex: Female.

Description: Figure 4 shows lower thoracic and upper lumbar vertebrae of a 65 year old female. We were unable to determine which thoracic vertebrae were involved here. Lytic lesions are present on the anterior of lower thoracic and upper lumbar vertebral bodies and have led to collapse of the spine. Healing has occurred through spontaneous fusion of vertebrae and posterior spinal elements near the site of collapse.

There was no indication of other diseases likely to have possibly caused the skeletal lesions (compression fractures, Paget's disease, osteomyelitis and neoplasms) in the medical records. Pulmonary TB was described and the destruction of the anterior region of the body of a single thoracic vertebra with resulting kyphosis indicates TB as the most likely cause of the lesions.

**Figure 5:**
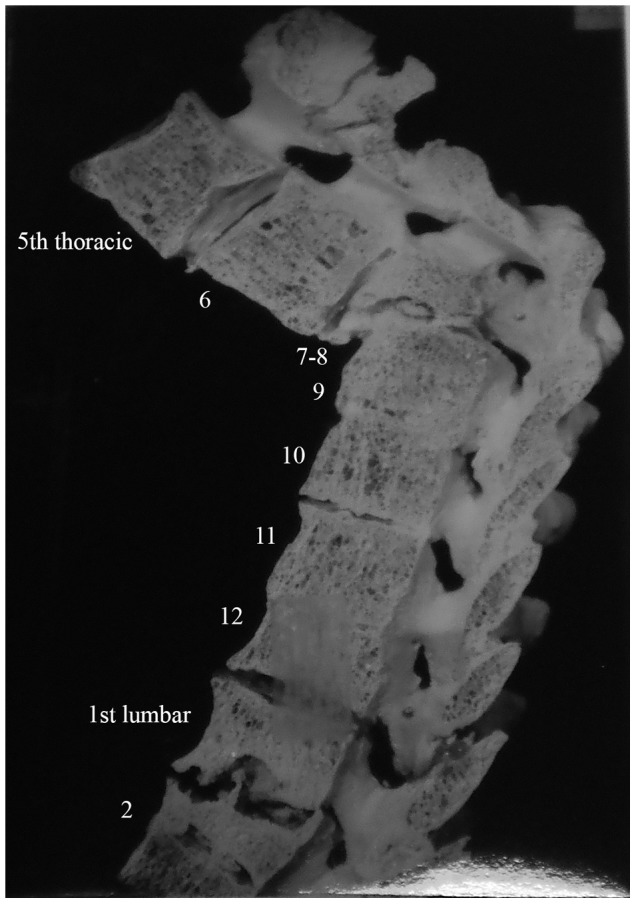
Autopsy Number: 901, Autopsy Year: 1948, Age: 52, Sex: Female.

Description: Figure 5 shows the fifth thoracic to the second lumbar vertebrae of a 52 year old female. Both thoracic vertebra seven and lumbar vertebra one were affected by TB spondylitis. The seventh thoracic vertebra has been completely destroyed, leading to collapse of the spine. The collapse of this vertebra resulted in a 90 degree angulation of the spine. Thoracic vertebrae six through twelve as well as lumbar vertebra one were all affected by the disease process. The spondylitis at the first lumbar vertebra has manifested as a deposition of bone on the superior surface of lumbar vertebra two which has caused destruction of the adjacent bone on the inferior surface of lumbar vertebra one. The bony protrusion is “pointed” and measures approximately 7 mm×2 mm. Healing has occurred by fusion of some posterior spinal elements. Interestingly, there is very little fusion between vertebral bodies, except between thoracic vertebrae eleven and twelve.

There was limited information regarding this individual in the medical records. It specifically details the presence of TB spondylitis of thoracic vertebra seven and lumbar vertebra one. Based on the specific destruction of the seventh thoracic vertebral body and resulting kyphosis, this diagnosis would be accurate.

#### Third time period (After 1950)

**Figure 6:**
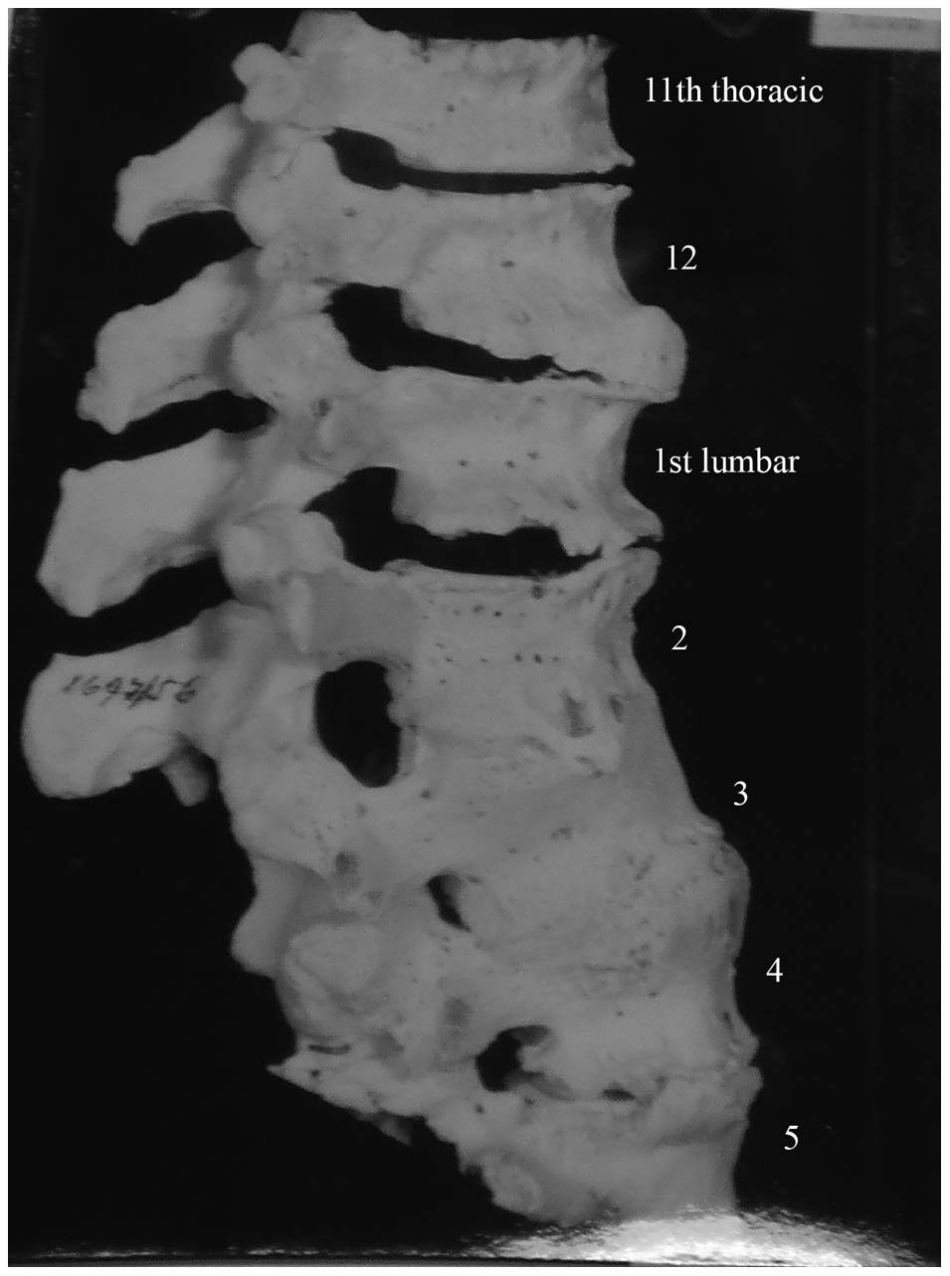
Autopsy Number: 1697, Autopsy Year: 1956, Age: 67, Sex: Female.

Description: Figure 6 shows the eleventh thoracic to fifth lumbar vertebrae of a 67 year old female. Lumbar vertebrae two to five have fused together through extensive bone deposition between vertebral bodies. There is evidence of lipping on the anterior edges of these vertebral bodies. Major lipping as a result of bone deposition is also present on the inferior edges of thoracic vertebra twelve and lumbar vertebra one. The TB spondylitis of lumbar vertebrae three to five occurred between 1933 and 1939; 20 years before death. This individual also had TB of the right knee and this became involved in 1949; 7 years before death. At autopsy, it was discovered that the knee was ankylosed and unable to be moved.

There was no mention of other diseases such as compression fractures, Paget's disease, osteomyelitis or neoplasms that might have been the cause of the skeletal lesions observed. The medical records describe the presence of TB spondylitis of the lower lumbar vertebrae as well as the knee. In this case, there is no kyphosis or collapse of the spine. There is an extensive amount of fusion, however this does not include posterior spinal elements. Due to the involvement of a single knee joint (TB is usually unilateral), it is likely that the spinal lesions are also caused by TB.

**Figure 7:**
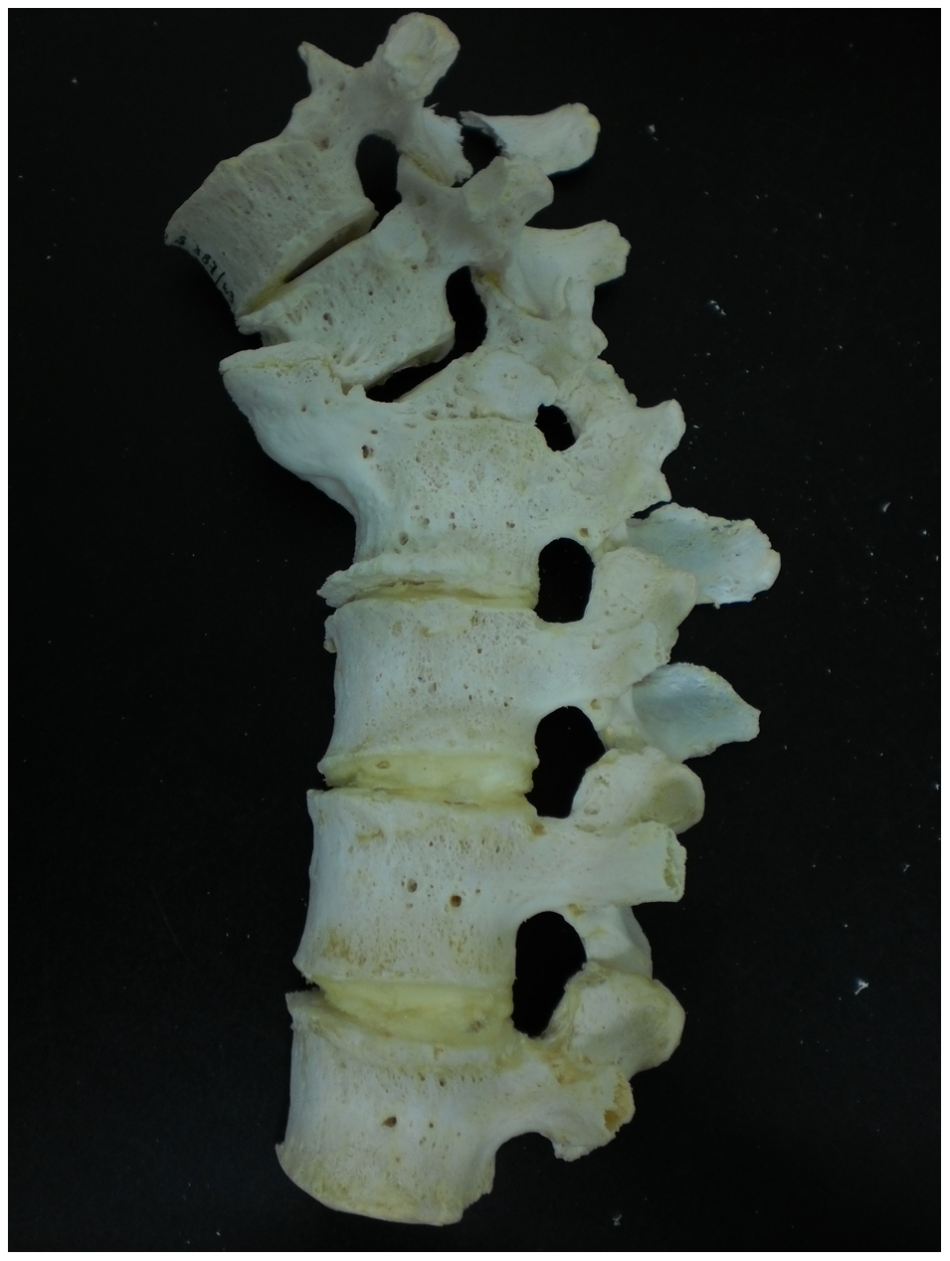
Autopsy Number: 387, Autopsy Year: 1963, Age: 52, Sex: Female.

Description: Figure 7 shows lower thoracic vertebrae of a 52 year old female. We were unable to determine which vertebrae are involved in this case. One of the vertebral bodies has been almost completely destroyed and the inferior surface has fused to the superior surface of the adjacent vertebra. There has been a large amount of bone deposition on the anterior edge of the vertebra below that which had collapsed; effectively creating a bridging structure. This extends across two vertebrae, skipping the one which had collapsed. Healing in this case is by bone deposition.

There was mention of neoplasms in the liver and iliac lymph nodes in this individual. There are details regarding the surgery attempted. The medical records also describe TB spondylitis of the thoracic vertebrae. One of the vertebrae has been almost completely destroyed, with the lytic lesion starting in the anterior region of the vertebral body. This has resulted in collapse of the spine. Posterior elements were unaffected. These are characteristics of TB rather than metastases and thus we considered TB the most likely cause of these lesions.

### Cases of spinal tuberculosis treated with surgically induced posterior spinal fusion

Four individuals from the Galler Collection were treated by this method. These are summarized below.

#### First time period (Before 1946)

**Figure 8:**
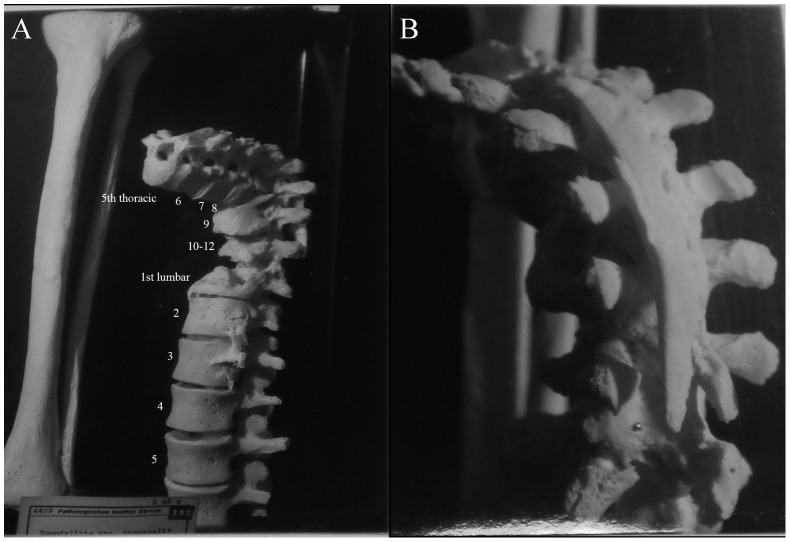
Autopsy Number: 7, Autopsy Year: 1928, Age: 30, Sex: Female.

Description: Figure 8 shows the fifth thoracic to fifth lumbar vertebrae of a 30 year old female. She had received a spinal operation for tuberculosis spondylitis, resulting in destruction of the anterior region of thoracic vertebrae ten to twelve. The spine has collapsed and spinal angulation had occurred. The lesions are completely destructive, however, a small mass of bone has been deposited on the left side of lumbar vertebrae one and two. This bone deposit measures approximately 22 mm×11 mm. No healing of the vertebral collapse is evident.

Since the autopsy of this case was carried out in 1928, there is limited information in the medical record. Spondylitis of the thoracic vertebrae due to TB was described. There was no mention of compression fractures, Paget's disease, osteomyelitis or neoplasms. However, based on the lesions observed, it is likely that TB is the cause. There is extensive destruction of the anterior regions of several thoracic vertebral bodies, leading to a collapse of the spine. The posterior elements were unaffected by the disease process, however, there was artificial fusion of the spine through surgical intervention.

#### Second time period (1946–1950)

**Figure 9:**
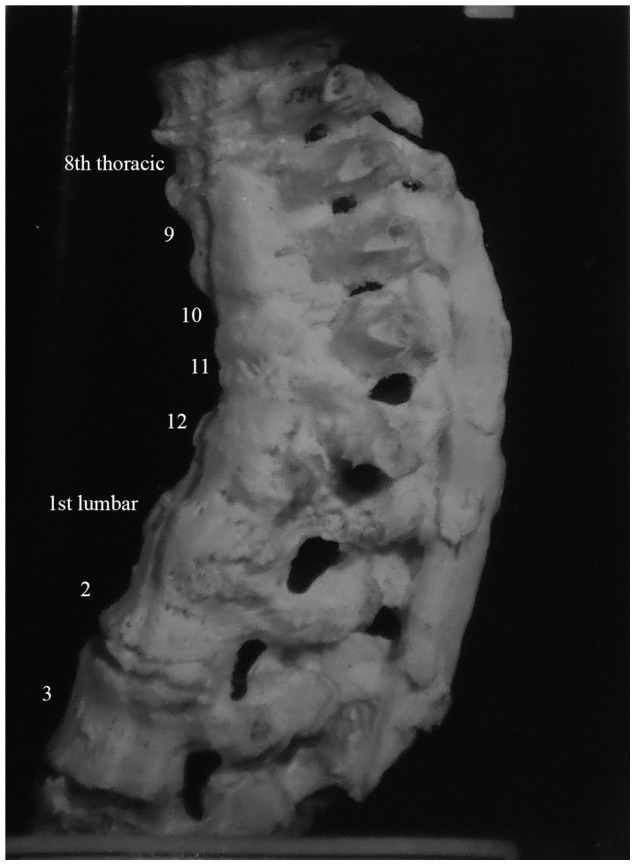
Autopsy Number: 262, Autopsy Year: 1948, Age: No information, Sex: No information.

Description: Figure 9 shows the eighth thoracic to third lumbar vertebrae of an individual with unknown age and sex. Extensive fusion on all edges (anterior, lateral and posterior) of thoracic vertebra nine to lumbar one has occurred. There is slight spinal angulation around thoracic vertebra eleven. This individual was treated with a spinal operation, 24 years before death and it was successful in that further spinal destruction has been avoided in this case. This operation was performed due to spondylitis of thoracic vertebrae eleven and twelve as well as lumbar vertebrae one to three. Healing has occurred spontaneously by bone block formation between vertebral bodies (fusion of vertebrae). Although bone deposition has occurred around posterior elements, upon examination, it was found these were not fused together. In this case, it is interesting to note that the level of bone deposition and vertebral fusion obscures any remaining destructive lesions, indicating this is a well healed case and the disease has been inactive for some time.

There is no mention in the medical records regarding compression fractures, Paget's disease, osteomyelitis or neoplasms, but there is a description of TB spondylitis. This case is more difficult to diagnose because of the extensive amount of bone deposition, atypical for TB. There is presence of a mild kyphosis and this is a case where TB was inactive for many years. Thus we can only diagnose this case as TB using information obtained from medical records, suggesting the possibility that these lesions may be the result of a condition other than TB. However, since this case involved artificial fusion of posterior spinal elements, it was not included in other analyses and therefore the inability to give a confident diagnosis does not affect our results.

#### Third time period (After 1950)

**Figure 10:**
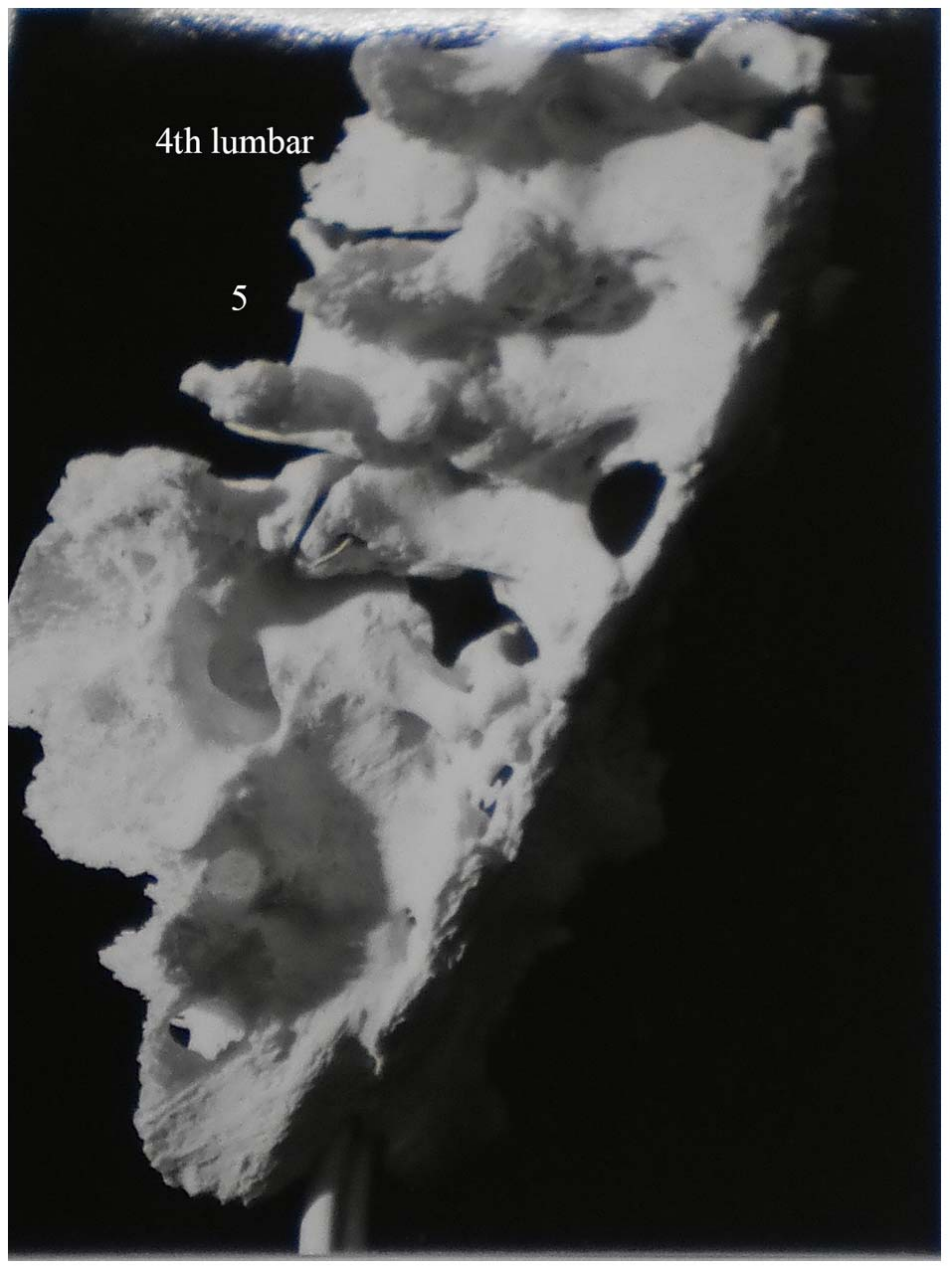
Autopsy Number: 779, Autopsy Year: 1953, Age: 50, Sex: Female.

Description: Figure 10 shows lumbar vertebrae four and five as well as the sacrum of a 50 year old female (anterior view). Minor destructive lesions are evident on the fourth and fifth lumbar vertebrae as the result of tuberculosis spondylitis starting 39 years before death, but this diagnosis was not made until 1925 (28 years before death). The individual was treated with a spinal operation and in this case, it has assisted in preventing massive destruction of the vertebrae. Both the diagnosis of spondylitis and the spinal operation occurred 39 years before death. Healing has occurred through a large amount of vertebral fusion including posterior elements. Lumbar vertebrae two to four are fused, four and five are fused into a square block and there is evidence of vertebral fusion of thoracic vertebra twelve to lumbar vertebra three. In addition to bony changes of the vertebrae, there was also extensive destruction of the sacrum.

The medical records describe spondylitis TB for this individual. No mention is made of compression fractures, Paget's disease, osteomyelitis or neoplasms. The lesions observed also indicate TB rather than another causative agent. There were lytic lesions on lumbar vertebrae and fusion of these vertebrae has resulted.

**Figure 11:**
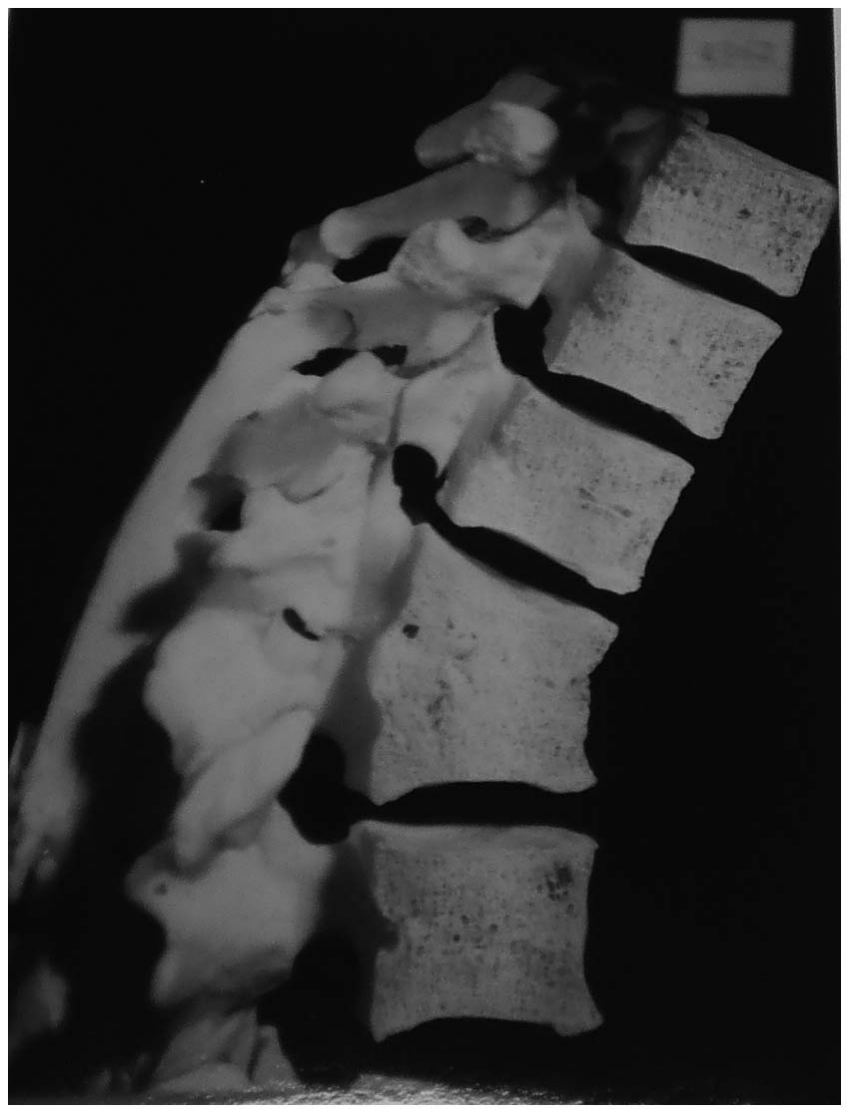
Autopsy Number: 5717, Autopsy Year: 1964, Age: 36, Sex: Female.

Description: Figure 11 shows thoracic vertebrae of a 36 year old female. We were unable to identify which thoracic vertebrae these are. A small, circular lesion is present on the thoracic vertebra at the bottom of Figure 11. It measures approximately 4 mm×3 mm. A spinal operation was performed eight years before death. Two vertebrae have fused spontaneously along the entire surface area of their vertebral bodies. Additional stabilization of the spine is provided by the surgical intervention.

Medical records describe spondylitis TB, but not compression fractures, Paget's disease, osteomyelitis or neoplasms. Only two thoracic vertebrae are involved, with destruction of the vertebral bodies, leading to a collapse of the spine. The two affected vertebrae have fused together. These are all characteristics of TB and thus we diagnosed this as a case of TB.

### Cases of skeletal tuberculosis not involving the spine

**Figure 12:**
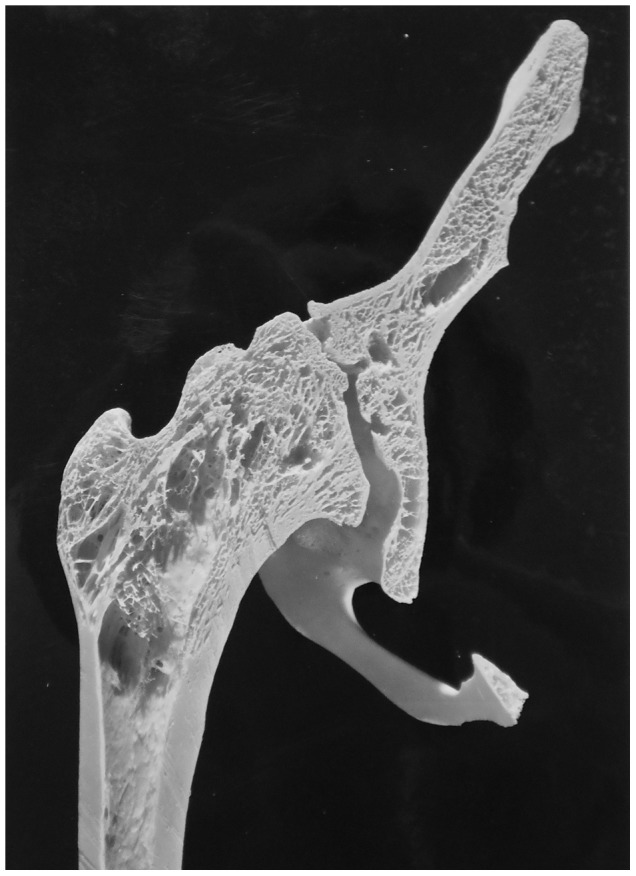
Autopsy Number: 325, Autopsy Year: 1936, Age: 16, Sex: Male.

Description: Figure 12 shows the right femur and acetabulum of a 16 year old male. There is extensive destruction of the articular surfaces of the hip joint. Although the individual had TB (beginning with meningeal) since 1924 (4 years old), bone destruction in the hip did not begin until shortly after he sustained an injury during 1929. Three years later, he returned to hospital. No evidence of healing is present.

We considered the possibility that these lesions could be the result of septic arthritis. Usually in septic arthritis, the bone destruction is limited and ankylosis very common [29], [30]. This case shows extensive destruction of both articular surfaces of the hip and no evidence of ankylosis. This individual was also very young and TB of the hip usually begins at an early age. Additionally, this case was from the first time period (before 1946) where antibiotics were unavailable. If the cause of these lesions were septic arthritis, it is likely this individual would have died before bone lesions could have developed [31]. This case was thus considered to be the result of TB.

**Figure 13:**
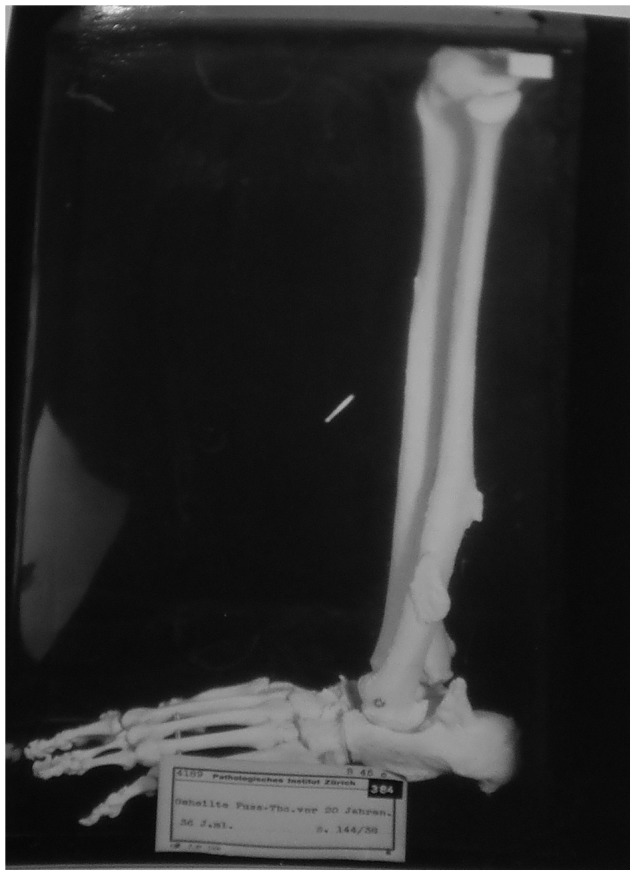
Autopsy Number: 144, Autopsy Year: 1938, Age: 36, Sex: Male.

Description: Figure 13 shows the foot bones and distal tibia and fibula of a 36 year old male. The individual also had skeletal TB of the metacarpal, elbow and left temporal bone since he was a child. These lesions, however, were not preserved in the collection. The final results of TB processes on the foot were healed by fusion of the fibula and tibia. This fusion had been going on for 20 years before death, indicating it was well healed and that the disease had been inactive for some time. The individual also received a rib resection and the right elbow is ankylosed to the point where it has become immobile.

This individual suffered from severe sepsis around the time of death. Due to this, the ankylosis of the elbow may be a result of septic arthritis rather than TB. However, for this study, we are more concerned with the bony changes in the foot because there is documented evidence of tuberculous diseases of this region. The medical records describe a well-healed example of foot TB and that other bones in the body were affected. Although the cause of death in this case would likely have been due to sepsis, TB occurred earlier in this person's life and the bone lesions are well healed at death. This indicates that sepsis was not the cause of these lesions, or healing would not have been observed.

**Figure 14:**
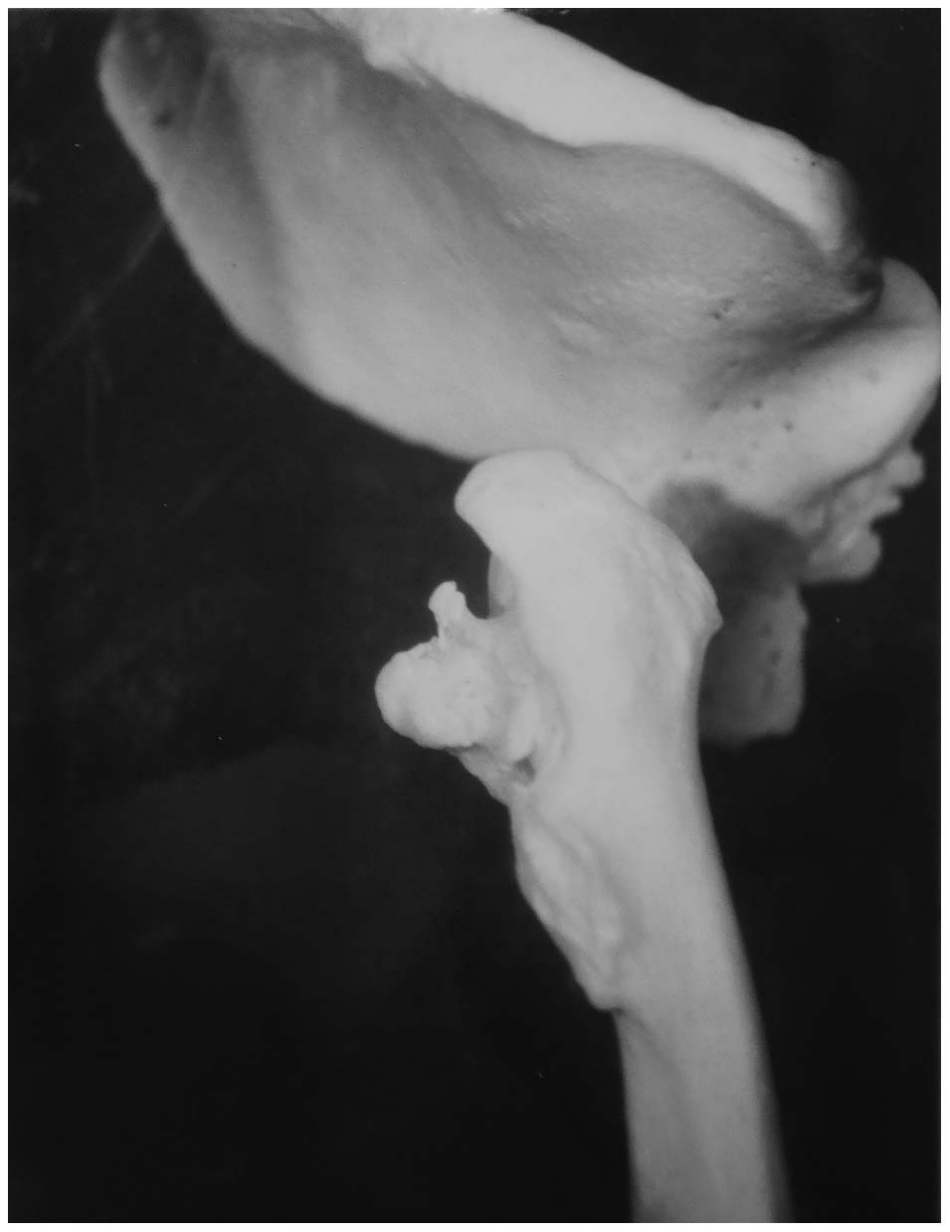
Autopsy Number: 304, Autopsy Year: 1947, Age: 37, Sex: Male.

Description: Figure 14 shows the right femur and acetabulum of a 37 year old male. There is a high level of destruction of articular surfaces of the femur and acetabulum due to TB infection. The femoral head is almost completely destroyed. The joint surface is mostly destroyed, however the joint is still mobile.

The diagnosis recorded in the medical records was one of two possibilities; TB or heart failure. Doctors were unable to decide. Although the cause of death could have been TB in this case, we are concerned with the etiology of the skeletal lesions of the hip. The medical records do clearly describe damage to the hip after infection with TB. The extensive destruction of the bone on the articular surfaces of the joint without ankylosis indicates TB is more probable than septic arthritis.

### Analysis of bone lesion healing

**Figure 15:**
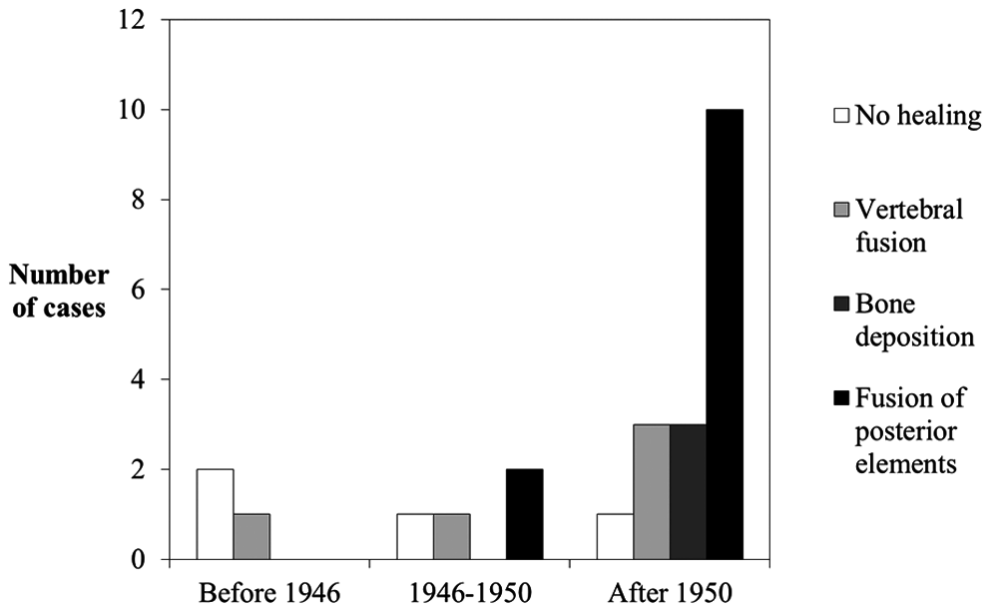
Number of cases from the Galler Collection for each category of healing of bone lesions due to tuberculosis through time (N = 25). Cases that received surgery (N = 4) were excluded.

There were two categories of lesion healing in the Galler Collection cases; the first followed targeted surgery for bone lesion repair and the second involved natural healing. The latter involved interventions not directly aimed at healing spinal lesions, but rather aiding the individual to combat the disease. The cases of artificial healing by posterior spinal fusion have been presented (N = 4), so we now focus upon the cases of natural healing (N = 25). The presence and extent of natural bone lesion healing in individuals from the Galler Collection are presented in Figure 15.

These results show a trend through time correlating with availability of pharmacological agents. Lesion healing in the first time period either did not occur or was achieved through fusion of vertebrae (which commonly occurs in skeletal TB). There were two cases where no healing of spinal lesions occurred and one with healing. However, in the second time period, where pharmacological agents (including antibiotics) were first being used to treat individuals, there are cases of healing involving fusion of posterior spinal elements. There was one case of no healing and three where healing had occurred. Further use and implementation of chemotherapeutic regimes resulted in all categories of bone lesion healing considered in this study, being present in samples from the third time period, including vertebral fusion, bone deposition, and fusion of posterior elements. In this time period, there was one case of unhealed skeletal lesions and 16 with healing.

Cases of TB affecting skeletal elements other than the spine were infrequent; there were only three cases in 69 individuals diagnosed with the disease. Two of these cases involved the hip and the last involved the foot. The cases affecting the hip joint show destruction of the articular surfaces; typical for TB. The individual with TB of the foot however, shows atypical lesions. The disease process is well healed and an extensive amount of fusion of bones has taken place.

The process of healing through vertebral fusion occurred throughout all time periods. There was no difference in appearance of lesions healed in this way when comparing examples throughout all time periods. Our results also show that different types of healing occur in later time periods (i.e. after the introduction of antibiotics), indicating that antibiotics did not affect the usual mechanism of healing (vertebral fusion), but did impact healing in general. A higher proportion of individuals in later time periods show healing than those in earlier time periods.

### Soft Tissue Involvement of TB

The number of sites affected by TB was recorded for each individual in the Galler Collection as either one (single) or more than one (multiple). The analysis involved comparison of single/multiple foci through time. For some individuals (N = 8), we were unable to determine a year of death and these cases were consequently excluded from this analysis.

**Figure 16:**
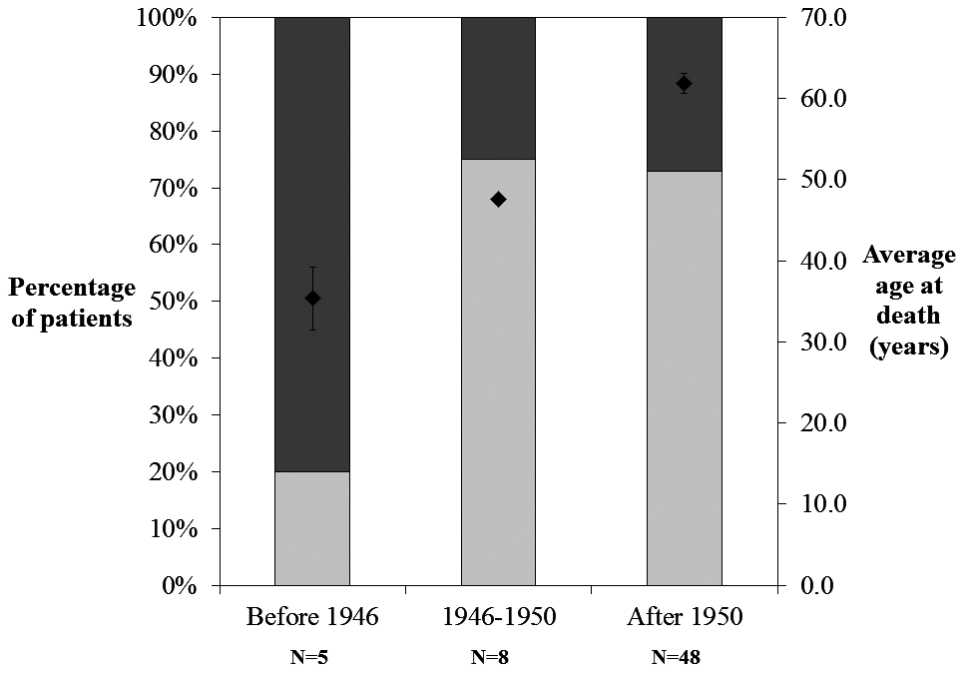
Percentage distribution through time of individuals from the Galler Collection with one region (single) and more than one region (multiple) of the body affected by tuberculosis. Average age of death (filled diamonds) is also plotted on the secondary (right) ordinate. Light and dark shading correspond to single and multiple foci, respectively.

The results (Figure 16) show that in the first time period, most individuals had multiple tuberculous foci. However, after the introduction of pharmacological intervention, the proportion of individuals with multiple foci decreased substantially. The third time period showed an essentially identical distribution to the second time period. The average age at death increased significantly through time.

It was possible to determine which regions of the body were affected by TB for every individual in the Galler Collection (N = 69), including those without observable skeletal lesions, using medical records (Table 2). Many individuals had TB in multiple tissues and bones of the body.

**Table 2:** Number of cases of tuberculosis affecting various soft tissues (and skeleton) of individuals in the Galler Collection (N = 69).

Tissue/bone	Number of cases	Percentage of total cases
Lung	36	52%
Spine	29	42%
Lymph nodes or vessels	9	13%
Urogenital	8	12%
Liver	7	10%
Other skeletal lesions (not already covered elsewhere)	7	10%
Spleen	5	7%
Suprarenal (adrenal)	5	7%
Hip	5	7%
Miliary	4	6%
Meningeal	3	4%
Elbow	2	3%
Knee	2	3%
Ear	1	1%
Gastrointestinal tract	1	1%
Aorta	1	1%
Psoas muscle	1	1%
Hand	1	1%
Tibia	1	1%
Feet	1	1%

Note that some individuals had tuberculosis of multiple tissues and bones.

Unsurprisingly, the most common site of TB was the lungs, closely followed by the spine. The lymph nodes/vessels, urogenital system, liver, skeleton, spleen, suprarenal glands and hip were affected in several cases (5–9 individuals each). Miliary (widespread) and meningeal TB affected fewer individuals; 4 and 3 cases respectively. Sites infrequently affected (1–2 cases each) included the elbow, knee, ear, gastrointestinal tract, aorta, psoas muscle, hand, tibia and foot. It was considered that there may be a link between regions of the body affected and skeletal lesions and this analysis will form a part of future studies of the Galler Collection.

Through time, the regions of the body affected by TB do not significantly change in frequency of infection and the lungs, spine, lymph nodes, etc. remain the main sites of pathology, regardless of the time period. There is a difference in the less frequently affected sites; all of the cases of TB affecting the meninges, elbow, aorta, psoas muscle, hand, tibia and foot occurred during the first time period.

A summary of the cases where multiple regions of the body were affected by TB is given in Table 3 and there are cases from each time period. The age range was 16 to 93 years, meaning that individuals of all ages were affected by TB in multiple tissues and bones of the body. The specific tissues affected varied between individuals and time periods but in all cases, the lungs were affected initially, though at the time of death lung pathology may not have been obvious and therefore not recorded.

**Table 3:** Summary of individuals with diagnosed tuberculosis of multiple regions of the body from the Galler Collection (N = 16).

Autopsy Number	Autopsy Year	Age (years)	Sex	Time Period	Soft tissues affected by tuberculosis
Data not available Patient ID: 404	Data not available	Data not available	Data not available	N/A	Skeleton (excluding spine), lung, urogenital, spleen and liver
793	1936	78	Female	Before 1946	Miliary: Lungs, urogenital, spleen, liver, suprarenal, aorta and hand
325	1936	16	Male	Before 1946	Meninges, hip, spleen and lungs
144	1938	36	Male	Before 1946	Skeleton (excluding spine), elbow, feet, tibia and meninges
751	1939	35	Male	Before 1946	Meninges, spine, lung, urogenital, psoas muscle,
304	1947	37	Male	1946–1950	Lung, lymph vessels and hip
779	1953	50	Female	After 1950	Spine, lungs, lymph nodes, suprarenals, liver and urogenital
1445	1954	88	Female	After 1950	Lung, lymph nodes, hip and knee
411	1955	34	Male	After 1950	Lung, lymph node, meninges, spine and urogenital
60	1956	69	Male	After 1950	Miliary: Spine, skeleton, lymph nodes, liver, spleen, suprarenal, genitourinary system and hip
1697	1956	67	Female	After 1950	Spine and knee
572	1957	93	Male	After 1950	Lungs, tracheal lymph node
487	1958	80	Male	After 1950	Lung and gastrointestinal system
749	1960	64	Male	After 1950	Skeleton (excluding spine), lung and spine
2420	1965	37	Male	After 1950	Spine and lungs
932	1966	72	Male	After 1950	Miliary: Lymph nodes, spleen and liver

### Co-morbidities

Many of the individuals in the Galler Collection had a number of other conditions in addition to TB. Table 4 shows the co-morbidities for all individuals with a year of death (N = 61) in each time period.

**Table 4:** Co-morbidities by time period associated with introduction and use of pharmacological interventions.

First period (before 1946)	%	Second period (1946–1950)	%	Third period (after 1950)	%
Fatty liver	60.0	***Pleuritis***	***25.0***	Heart pathology	48.2
***Pleuritis***	***40.0***	None	25.0	Arterio/atherosclerosis	39.3
***Pneumonia***	***40.0***	Heart pathology	12.5	Osteoporosis	37.5
Edema	40.0	Arterio/atherosclerosis	12.5	Edema	35.7
Peritonitis	40.0	Osteoporosis	12.5	***Pneumonia***	***21.4***
Tonsilitis	40.0	***Pneumonia***	*12.5*	Emphysema	19.6
None	20.0	***Underweight***	*12.5*	Bronchitis	19.6
***Underweight***	***20.0***	Metastases	12.5	***Underweight***	***17.9***
Emphysema	20.0	Fatty liver	12.5	***Pleuritis***	***16.1***
Sepsis	20.0	Congenital hip luxation	12.5	Metastases	10.7
Addison's disease	20.0	Blood disease	12.5	Cystitis	10.7
		Aortic aneurism	12.5	Uremia	10.7
				Senile marasmus	10.7

Values reflect the percentage of total individuals in that time period with a specific disease or condition. *Highlighted in bold and italics are causes related to TB that consistently appear in all time periods.*

Across all time periods, there are several co-morbidities that were common among all groups. These include pleurisy, pneumonia and being underweight. Additionally, the effect of antibiotics can be observed between groups. In the first time period, infectious diseases are included in the co-morbidities list. However, in the second and third time periods, the co-morbidities become chronic and “degenerative” in nature, reflecting a shift away from infectious diseases due to the introduction of pharmacological agents. Several chronic co-morbidities from the second and third time periods include heart pathologies, osteoporosis and metastases (cancer).

## Discussion

This study showed that “healing” of spinal lesions due to TB can be achieved through the processes of vertebral fusion, bone deposition and fusion of posterior elements. We also show one example of healed foot TB where bones have become extensively fused together. Although medical records may be inaccurate due to difficulty in diagnosis, we also considered skeletal evidence in each case. We did encounter difficulties with incomplete skeletons in the collection. However, in some cases, the medical records did provide additional information regarding bones that were not present. Through a combination of skeletal evidence and medical records, we were able to confidently suggest that all individuals presented here except for three (of 29) had skeletal lesions as a result of TB. Consequently, the results presented here can be of use to palaeopathologists when considering skeletal samples with bone lesions suggestive of TB, but with some atypical characteristics.

The small sample size (N = 25) is a major limitation of this study, but can be addressed by reviewing published literature regarding similar findings in the past. Several studies have previously described cases of diagnosed spinal TB from the pre-antibiotic era [9], [32], [33]. In two of these studies [32], [33], none of the individuals received pharmacological treatment but bone deposition did still occur (Table 5). Individuals were aged between 18 and 39 years. Each case was diagnosed as TB using biopsy, smear tests and inoculation of guinea pigs. The ancestry of each individual was noted in Table 5 because the living standards in the United States at the time the studies were conducted (early 20^th^ century) were different for those with European compared with African-American ancestry [34]. It has also been noted that African-Americans as well as American Indians appear to have a higher risk of developing active TB than Europeans [35]. Despite these differences, bone deposition, defined in these studies as “bony bridging,” was noted in several individuals of different ancestry. An estimate of the frequency of “bony bridging” (recorded from the Cincinnati General Hospital, Ohio) in the early 20^th^ century was 10% in all spinal cases of TB [32]. This value is the same as that reported by [10] for 1922. In another study by [6], it was reported that the use of antibiotics increased the percentage of cases healing through bony fusion to 50%. In another separate study, a similar observation was found from 44% (no antibiotics) to 55% (antibiotics). “Superior healing” has also been described in cases treated with streptomycin compared to those who were left unhealed [9], [36]. Finally, spinal fusion was found to be 69% in a series of cases treated with streptomycin, while fusion was 31% in a comparable sample that was untreated [37].

**Table 5:** Summary of cases of spinal tuberculosis (TB) from the pre-antibiotic literature for comparison with this study.

Reference	Country	Ancestry	Age (years)	Sex	Date of admission	Date of release or death	Disease focus	Bone lesions	Treatment
(Perlman and Freiberg 1943)	United States	European	32	Male	May 6, 1940	December 12, 1940 (death)	Spinal TB affecting lumbar vertebrae	Disc space between L2 and L3 is narrowed, a calcified deposit bridges this space	Bed rest at the hospital
(Perlman and Freiberg 1943)	United States	African American	18	Male	April 9, 1937	June 1940 (released)	Pulmonary, lumbar spine, psoas muscle and right hand	Destruction of L5, dense bony bridging between L3/L4 and L4/L5, sacroiliac involvement	Not specified
(Perlman and Freiberg 1943)	United States	African American	32	Male	August 8, 1937	February 13, 1938, readmitted on April 17, 1940	Pulmonary, lumbar spine, lymph nodes, both psoas muscles	Gibbus of L4/L5, destruction of L4/L5, bony bridging between L3/L4	Not specified
(Perlman and Freiberg 1943)	United States	European	25	Male	January 9, 1941	Not specified	Lower lumbar, left hip, psoas muscle, lymph node	Bony bridging between L1/L2	Not specified
(Perlman and Freiberg 1943)	United States	European	39	Male	July 14, 1939	Not specified	Left hip, lumbar spine, psoas muscle, lymph nodes	Intervertebral disc between L2/L3 destroyed, bony bridging between L2/L3, L3/L4 and L4/L5	Surgery on left hip
(Cofield 1922)	Italy	European	24	Male	November 1917	Not specified	Lumbar spine, psoas muscle	Bony bridging between L2/L3	

Note that “L1” refers to lumbar vertebra number one and “L2” to the second lumbar vertebra, etc.

Our suggestion for the reason these types of bony changes occurred in individuals from the Galler Collection is due to elimination or complete control of the bacterium through a combination of an individual's immune system and treatment by pharmacological intervention (including antibiotics). Although this may not result in complete removal of the bacterium from an individual's body, it may be sufficiently controlled by granulomatous tissue to render it “neutralized.” [6] observed that destructive processes can be stopped in as little as 6 to 9 months with antibiotics. After cessation of those processes involving the bone, other changes can occur. In the cases where a longer time period has passed since the disease was active, bone may be deposited to provide strength to a spinal column weakened by bone destruction. We found this to be true in at least three cases, where the disease had been arrested and bone deposition had occurred over a long time span. Bone deposition and fusion has been found to take a long time; usually several years [38]. However, it has been reported that bone solidification, fusion and ankylosis is an outcome for TB affecting the skeleton [11], [38]. [6] has reported the ankylosis of cases of TB of the hip and knee that were only treated using immobilization techniques. The same authors have also reported a large proportion of lumbar vertebrae (66%) lesions progressing to ankylosis and bony fusion. Additionally, fusion of posterior spinal elements such as the spinous process can also provide physical support in an area around a collapsed vertebra. [6] also made this observation in patients who were recommended for posterior spinal fusion. Upon surgery, it was discovered that in some, spontaneous fusion of the vertebrae at the apex of the kyphosis has occurred. Some individuals (from all time periods) from our study as well as other studies from the literature received spinal operations. While conservative therapy such as rest, hygiene and improved nutrition can produce satisfactory results in the treatment of TB, this takes a considerable amount of time [39]. Surgery can offer a faster and more effective means of treatment [40]. The specific surgery we observed has been very popular and successful in the past. [39] report on five cases treated with posterior spinal fusion for correction of kyphosis and prevention of further deformity in the spine. All patients survived this operation and it was successful in preventing further deformity.

Healing of TB lesions occurs through the processes of fibrosis, organization of abscesses, resorption of sequestra, osteogenesis and occasionally bony fusion between vertebrae [33]. In Pott's initial report of TB spinal lesions, he described calcification of intervertebral ligaments and production of new bone but most clinicians consider these elements not diagnostic of the disease [32], [33]. Spinal fusion is most likely to occur when kyphosis has caused adjacent vertebrae to come in contact with one another [38]. Bone can react in only a limited number of ways to pathological processes and is heavily influenced by a number of factors including the presence of other infectious agents or pathology, the immunity of an individual, genetics of the pathogen and location of the lesion [32]. [6] also suggests that there may be a difference in healing when certain antibiotics are used. Work on renal TB showed that when streptomycin and PAS were used, healing occurred by fibrosis. When streptomycin and isoniazid were used, lesions healed with minimal scarring. These problems however, do not affect our results because we collected data from historical records, medical and autopsy reports in addition to skeletal samples. The medical reports may be considered to be accurate because TB was well described by the 20^th^ century in Europe and a number of routine diagnostic techniques (e.g. culture, guinea pig inoculation, smear tests) were available at this time [41].

A comparison of descriptions published regarding palaeopathological cases of TB with our cases was made. We included only several examples from the newer literature because these are more likely to have very extensive descriptions which will allow the best comparison. We chose recent articles [12], [42]–[45] as these represent some newer cases from various geographical regions (Hungary, Peru, Siberia and Korea). The cases from Peru described by [42] all showed typical destructive lesions for TB, with no new bone formation. [45] and [43] both report on spines with extensive amounts of vertebral fusion from Korea and Hungary, respectively. However, they note that no new bone was formed, despite the large amount of fusion. In [44], many of the cases from Siberia show destructive lesions, but in one individual (XXXI.77, Figure 3 of publication), some bone deposition is present on the edges of two vertebral bodies. Finally, [12] report on several cases from Hungary and one of these (Grave No.: 39/02 (9th century), Inventory no.: 2008.4.118., Figure 8) had lesions very similar to one of our cases (Autopsy Number: 732, Autopsy Year: 1949, Figure 4). Several separate foci with vertebral fusion were present, along with bone deposition on a lumbar vertebra. While some of our cases do show a lack of bone formation, many show bone deposition, similar to the last example from [12] above. Therefore, our results may be useful for applications in paleopathological studies. Where some cases display bone deposition atypical for TB (spinal or otherwise) that would automatically exclude the disease from differential diagnoses, these observations may assist to identify unusual cases. Our results may also help to initiate further investigations such as ancient DNA [12], [42], [44], [46] or lipid analysis [47]–[50] of skeletal remains that would otherwise not be considered as potentially useful samples for TB studies. This may be useful since these methods are expensive and time consuming.

Another factor to consider in this study is the way in which the individuals were divided into time periods. TB is a chronic disease with both active and latent periods [8]. Older individuals may have developed TB during the first time period, but died only in the third time period, complicating the results. However, if the individual survived until antibiotic treatment was available, then they would experience healing after control of the bacterium, same as another individual who developed skeletal lesions of TB during the third time period would. Additionally, when the Swiss Law [28] was introduced, it required the compulsory reporting and treatment of TB cases. As such, when antibiotics were introduced, it can be expected that in a wealthy country such as Switzerland with excellent healthcare, individuals with TB would be treated with these pharmaceutical agents. A study in Minnesota, United States during 1946–1948 reported the rates of streptomycin usage [37]. In 1946, only 5% of patients received streptomycin. In 1947 and 1948, the rates were 52% and 43%, respectively. Since streptomycin was only introduced in 1946 [19], this is a rapid adoption of its use into general practice. Therefore we can consider that *at least* half of the individuals in our collection who died after 1946 were treated with antibiotics. This allows a generalization of the effects of streptomycin on skeletal lesions.

Analysis of the number of tuberculous foci after the introduction and use of pharmacological agents to treat Swiss individuals during the time period 1946–1977 showed that an individual's prognosis was improved when these agents were used in treatments. [6] has also reported a decrease of multiple foci after streptomycin was introduced. The reduction was 50%; from 10% to 5%. While these values do not agree with our findings this can be explained by differences in the samples. Individuals in the Galler Collection are a small sample of adults with exemplary skeletal lesions, while the sample described by [6] is a generalization of the South African population and includes all ages, whether they have skeletal manifestations or not. Another study [36] reported a positive general effect on the immunity of patients. They report that although streptomycin was unable to reach the site of a lesion directly, the benefits to the immune system allowed a patient to combat the bacterium more effectively. This resulted in cessation of the destructive processes. The results for the regions of the body affected by TB in individuals from the Galler Collection agree with previous reports of tuberculous involvement of soft tissues and bone. TB is primarily a pulmonary disease and it is expected that the most frequent site of infection would be the lungs, which is supported here. Other soft tissues involved in the immune response are also frequently affected in individuals from the Galler Collection, such as the liver and spleen. Skeletal TB makes up 10.1% of the cases and these involve the spine, elbow, hip, knee, tibia and feet. Several of these sites are commonly reported as major sites of tuberculous involvement in active disease (hip and knee, specifically). Additionally, the gastrointestinal tract is affected in only a single case, suggesting that the primary mode of transmission is through inhaled aerosolized droplets generated by coughing although abdominal TB can be transmitted from cattle by ingestion of contaminated milk and meat products [51]. Additionally it was observed that the main sites of tuberculous involvement (lungs, spine, lymph nodes, etc.) remained consistent through time. However, there were additional cases of TB of infrequently affected sites (meningeal, elbow, aorta, psoas muscle, hand, foot and tibia) during the first time period. The reason for this change in disease manifestation may be due to the implementation of pharmacological treatment during later time periods. Without drug therapy, an individual's immune system was not supported by medical means and consequently the disease was able to spread to areas of the body that are infrequently involved in TB. A summary of the cases with TB of multiple regions of the body showed no trends associated with age or time period. Individuals from all time periods were affected, showing that the implementation of pharmacological therapy regimes did not completely prevent an individual from developing TB in multiple tissues and bones of the body.

The co-morbidities observed to be common to all three time periods (underweight, pneumonia and pleurisy) were expected. TB often causes a loss of appetite leading to weight loss and consequently underweight individuals [2], [40]. Pneumonia and pleurisy are both conditions affecting lung tissues and this may reduce an individual's immunity to pulmonary TB. The increase in chronic, degenerative conditions through time indicates that antibiotics were an effective treatment for infectious diseases and that individuals were living long enough with TB to develop these diseases. This is also reflected in an increase in the average age at death through time. Despite these additional co-morbidities, most patients can be expected to recover after some time. This is due to antibiotics as well as other treatments such as rest as well as improved nutrition and hygiene [6].

## Conclusions

This study showed that “healing” of osteolytic TB lesions in both the spine and other regions of the skeleton occurred during the 20^th^ century in Switzerland. This may occur through fusion of anterior and posterior parts of vertebrae, bone deposition and was aided by surgical intervention (e.g. posterior spinal fusion). Although bone lesion healing occurred naturally prior to the introduction of pharmacological interventions, our results suggest an increase in the frequency and effectiveness of healing after the introduction of pharmacological agents. This information may be used to aid differential diagnosis in unusual paleopathological cases of TB.

## Supporting Information

Figure S1 **Autopsy Number: 739, Autopsy Year: 1948, Age: 76, Sex: Female.**
(TIF)

Figure S2 **Autopsy Number: 60, Autopsy Year: 1956, Age: 69, Sex: Male.**
(TIF)

Figure S3 **Autopsy Number: 487, Autopsy Year: 1958, Age: 80, Sex: Male.**
(TIF)

Figure S4 **Autopsy Number: 1441, Autopsy Year: 1954, Age: 80, Sex: Male.**
(TIF)

Figure S5 **Autopsy Number: 2420, Autopsy Year: 1965, Age: 37, Sex: Male.**
(TIF)

Figure S6 **Autopsy Number: 1219, Autopsy Year: 1969, Age: 69, Sex: Female.**
(TIF)

Figure S7 **Autopsy Number: 785, Autopsy Year: 1963, Age: 64, Sex: Male.** Note that Figure 1D also shows another image of this case.
(TIF)

Figure S8 **Autopsy Number: 1167, Autopsy Year: 1960, Age: 94, Sex: Female.**
(TIF)

Figure S9 **Autopsy Number: 1227, Autopsy Year: 1969, Age: 89, Sex: Female.**
(TIF)

Figure S10 **Autopsy Number: 1466, Autopsy Year: 1966, Age: 85, Sex: Female.**
(TIF)

Figure S11 **Autopsy Number: 1485, Autopsy Year: 1960, Age: 31, Sex: Female.**
(TIF)

Figure S12 **Autopsy Number: 1959, Autopsy Year: 1966, Age: 76, Sex: Female.**
(TIF)

Figure S13 **Autopsy Number: 2289, Autopsy Year: 1968, Age: 60, Sex: Male.**
(TIF)

Figure S14 **Autopsy Number: 2461, Autopsy Year: 1969, Age: 69, Sex: Female.** Note that Figure 1C also shows another image of this case.
(TIF)

Appendix S1 (PDF)
