# Supplementary figures and images for: Skeletal Lesions in Human Tuberculosis May Sometimes Heal: An Aid to Palaeopathological Diagnoses
Source: PLoS One. 2013 Apr 24;8(4):e62798. doi: 10.1371/journal.pone.0062798 (PMC3634763; doi:10.1371/journal.pone.0062798)

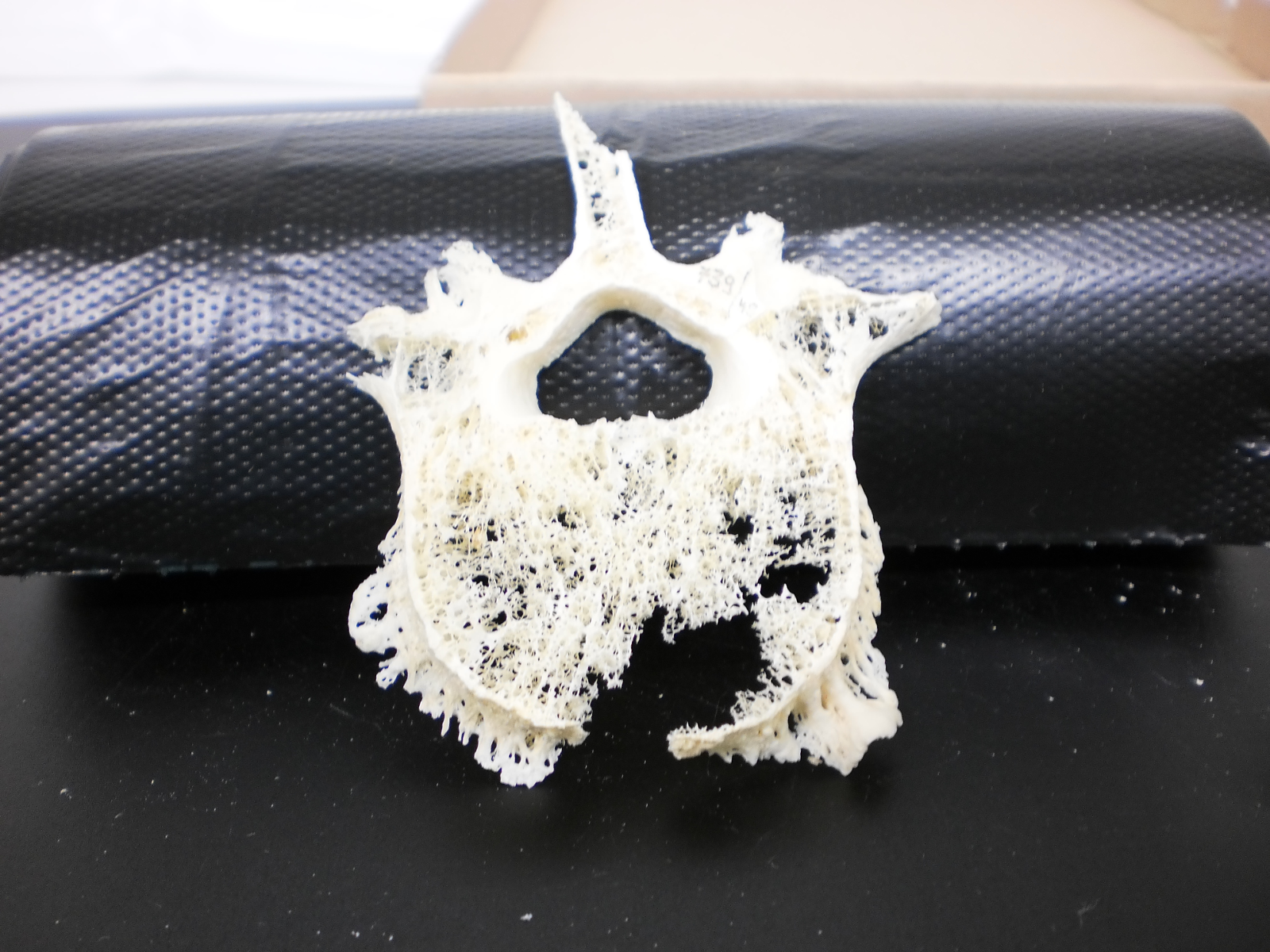

Supplement: Figure S1 — Autopsy Number: 739, Autopsy Year: 1948, Age: 76, Sex: Female. (TIF) [file pone.0062798.s001.tif]

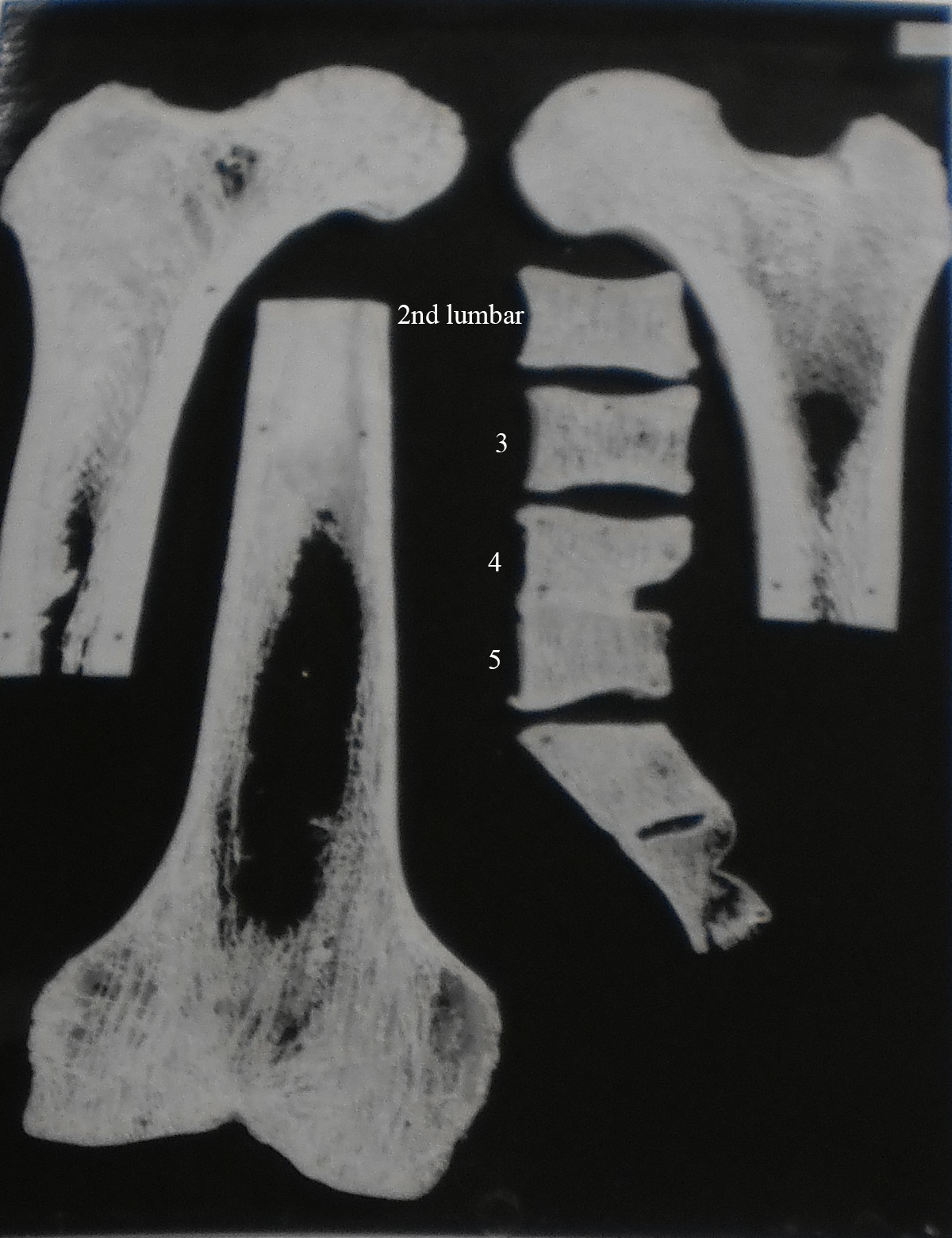

Supplement: Figure S2 — Autopsy Number: 60, Autopsy Year: 1956, Age: 69, Sex: Male. (TIF) [file pone.0062798.s002.tif]

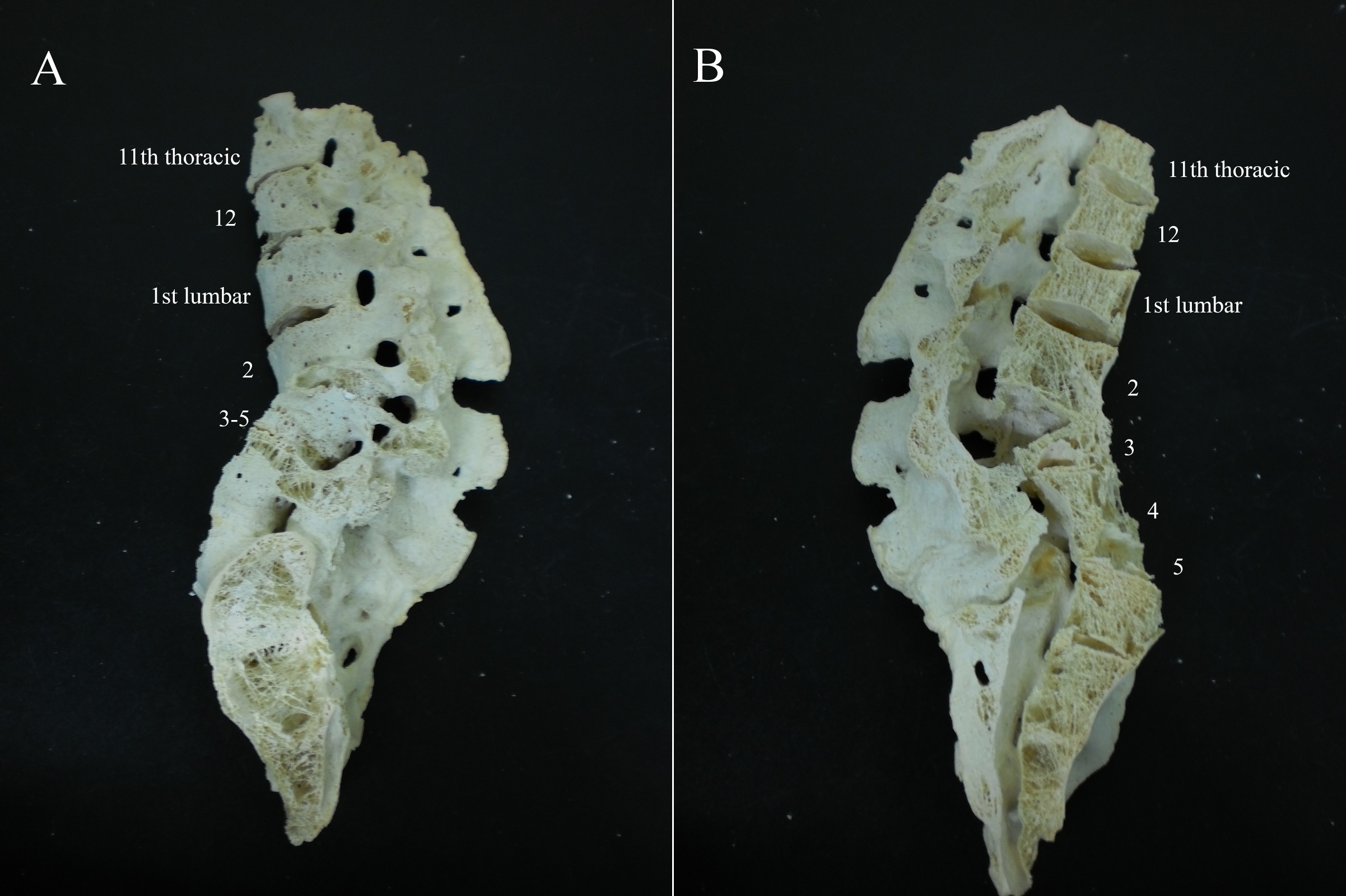

Supplement: Figure S3 — Autopsy Number: 487, Autopsy Year: 1958, Age: 80, Sex: Male. (TIF) [file pone.0062798.s003.tif]

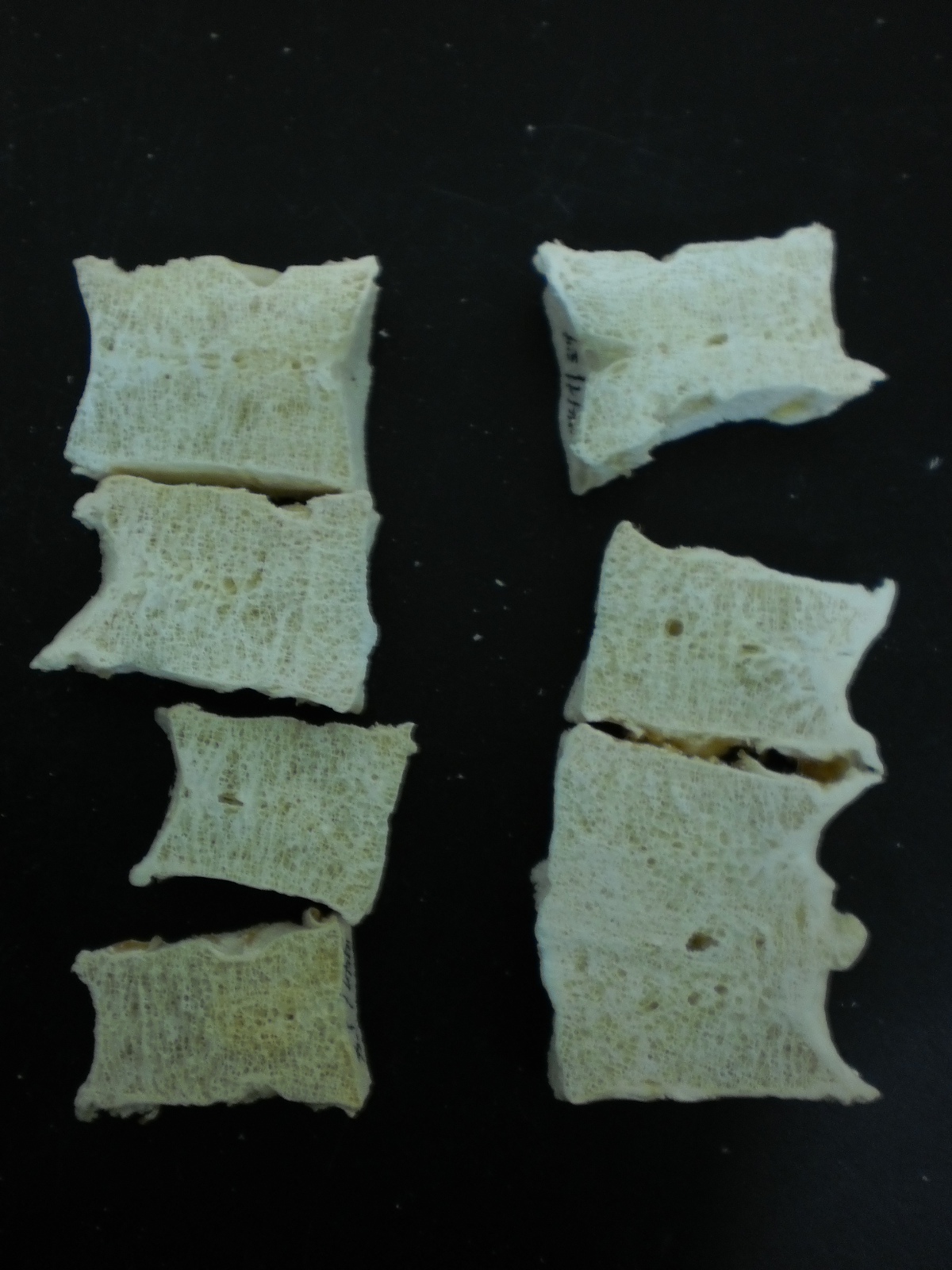

Supplement: Figure S4 — Autopsy Number: 1441, Autopsy Year: 1954, Age: 80, Sex: Male. (TIF) [file pone.0062798.s004.tif]

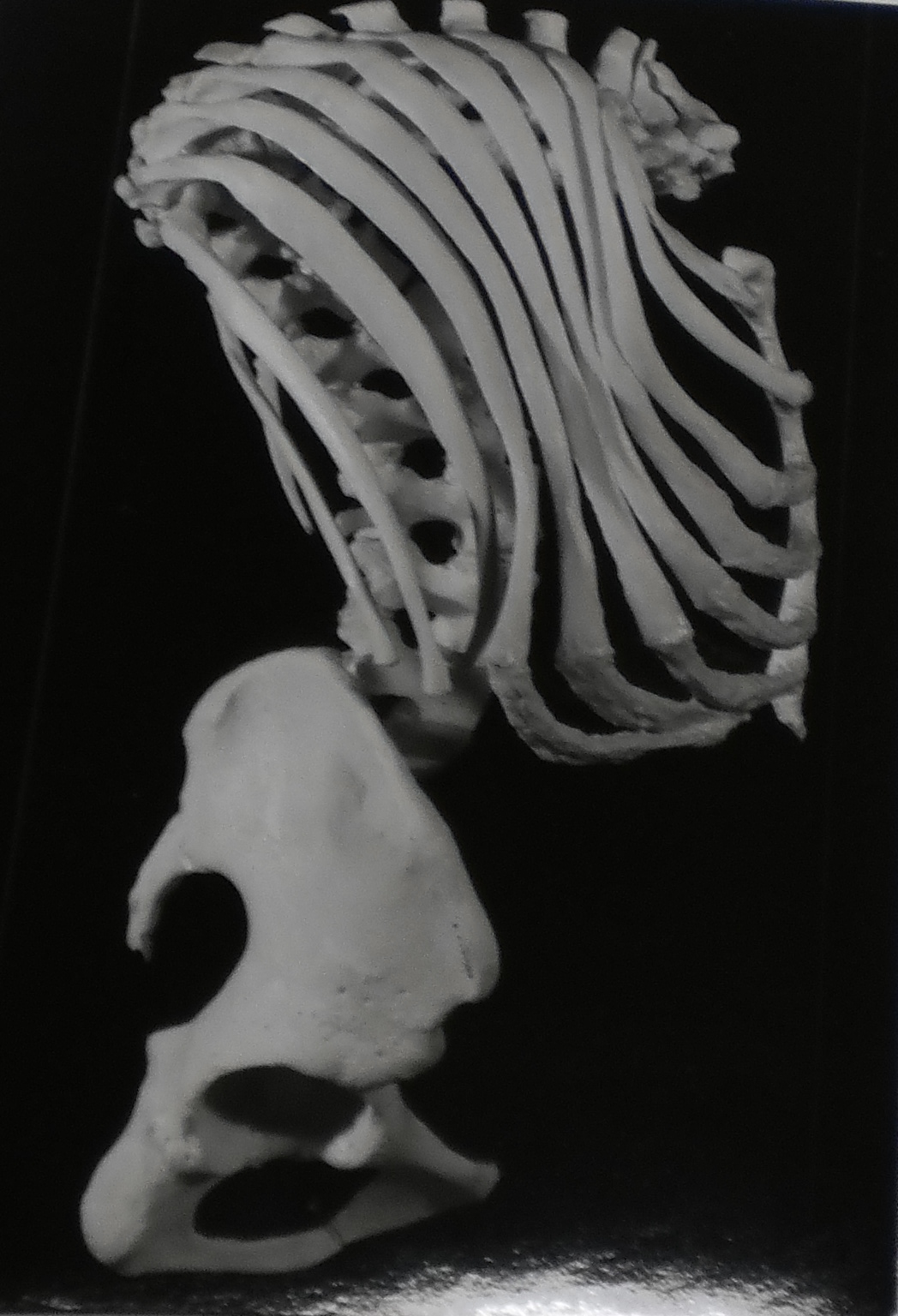

Supplement: Figure S5 — Autopsy Number: 2420, Autopsy Year: 1965, Age: 37, Sex: Male. (TIF) [file pone.0062798.s005.tif]

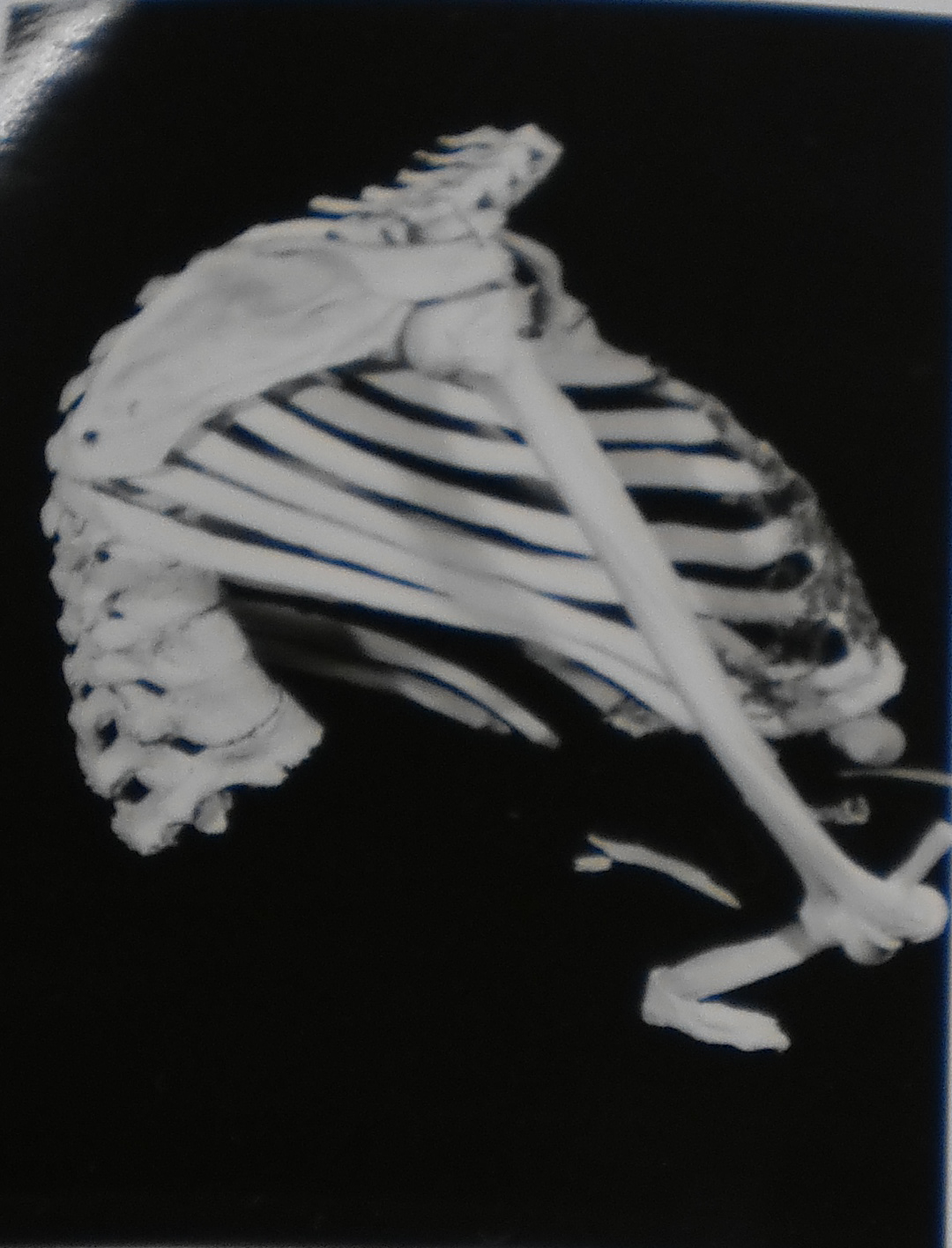

Supplement: Figure S6 — Autopsy Number: 1219, Autopsy Year: 1969, Age: 69, Sex: Female. (TIF) [file pone.0062798.s006.tif]

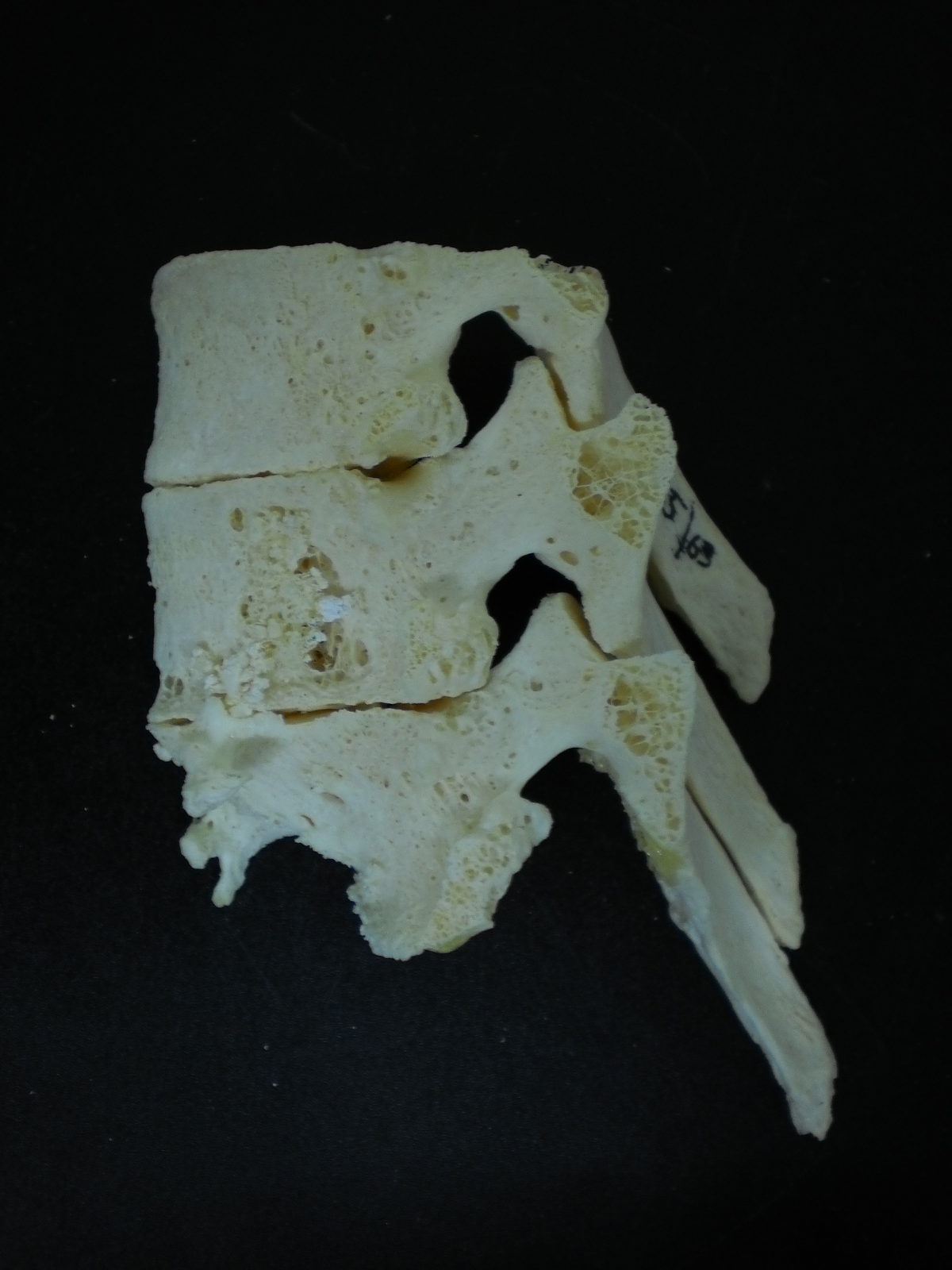

Supplement: Figure S7 — Autopsy Number: 785, Autopsy Year: 1963, Age: 64, Sex: Male. Note that Figure 1D also shows another image of this case. (TIF) [file pone.0062798.s007.tif]

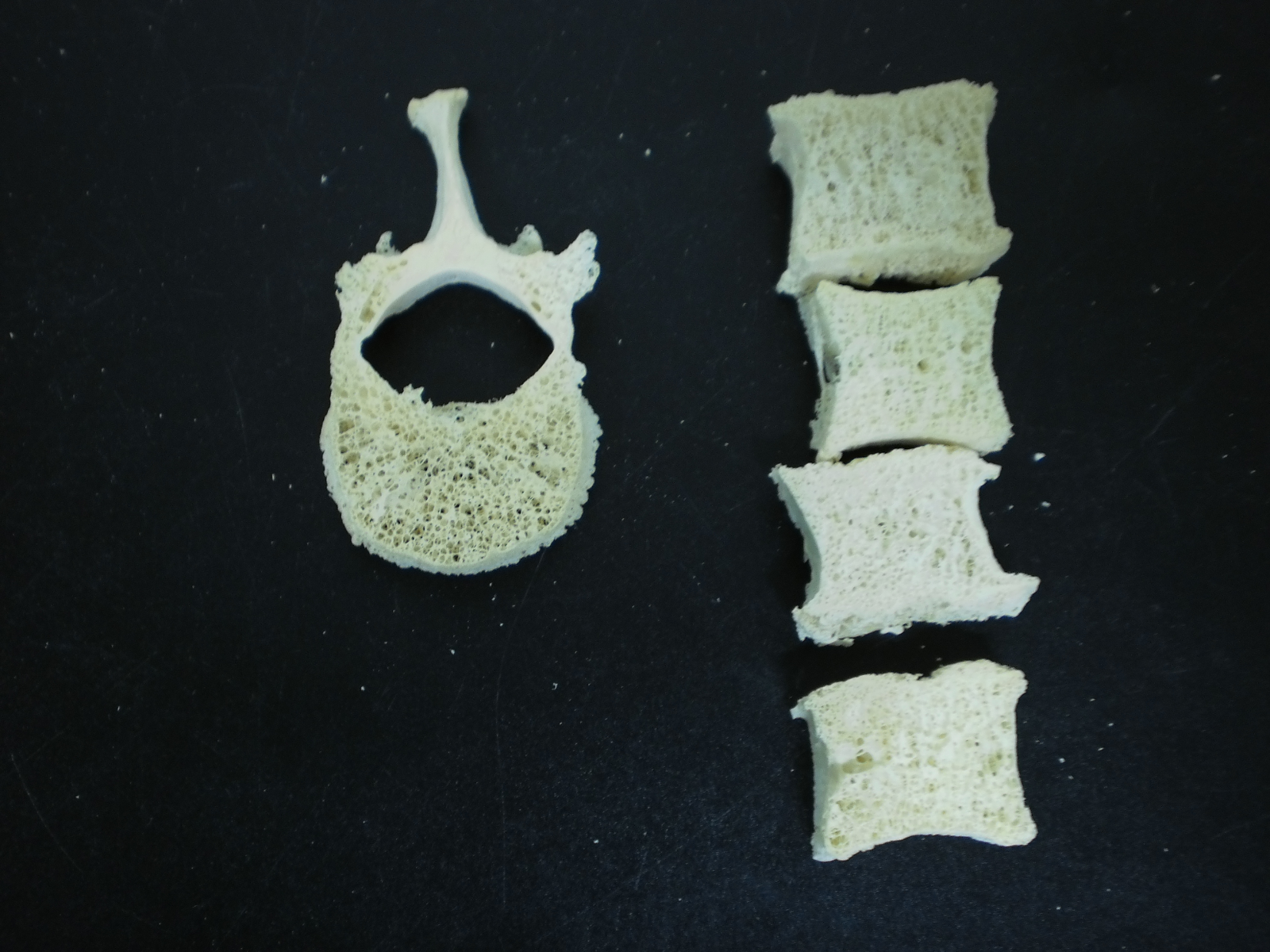

Supplement: Figure S8 — Autopsy Number: 1167, Autopsy Year: 1960, Age: 94, Sex: Female. (TIF) [file pone.0062798.s008.tif]

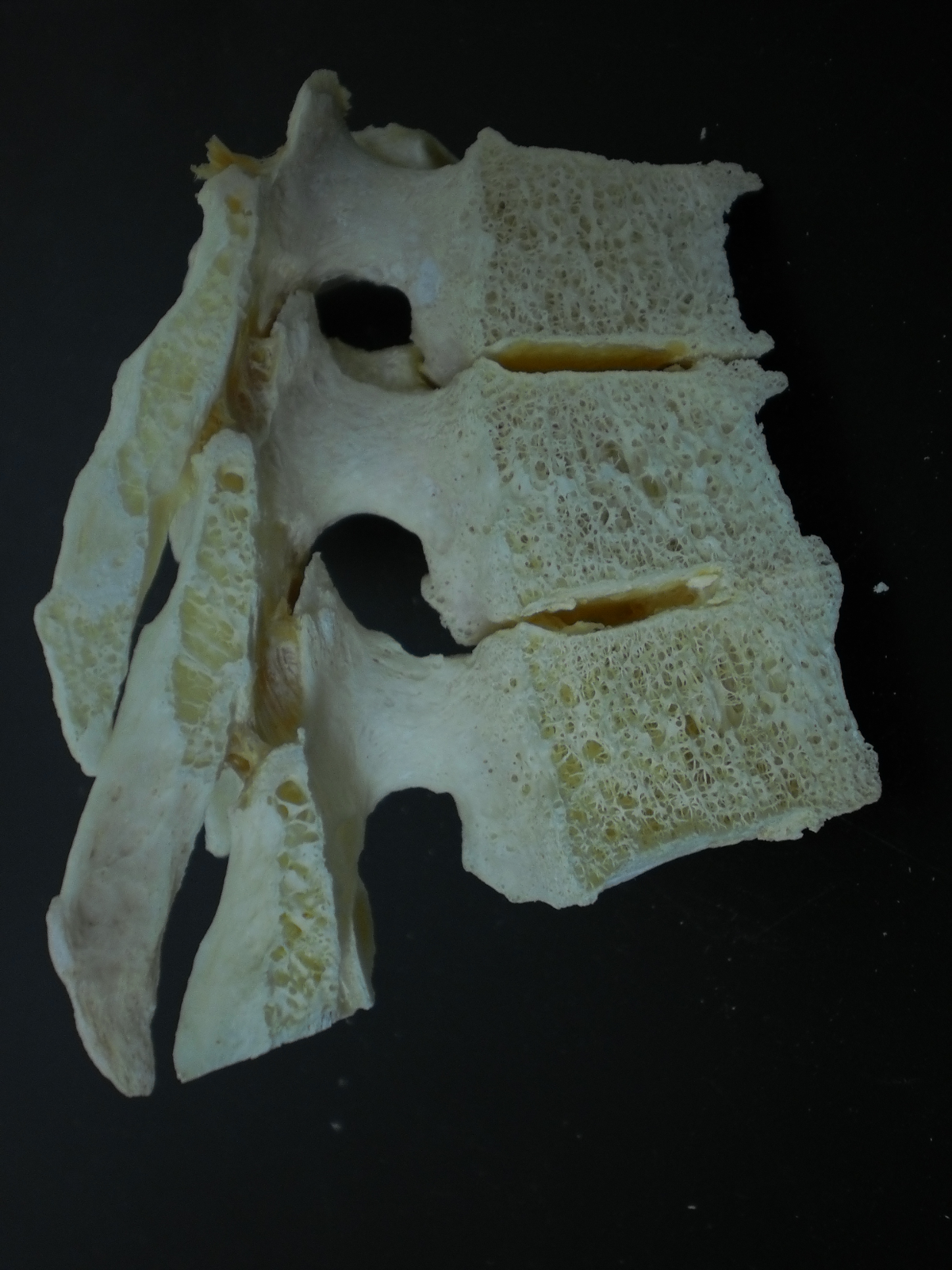

Supplement: Figure S9 — Autopsy Number: 1227, Autopsy Year: 1969, Age: 89, Sex: Female. (TIF) [file pone.0062798.s009.tif]

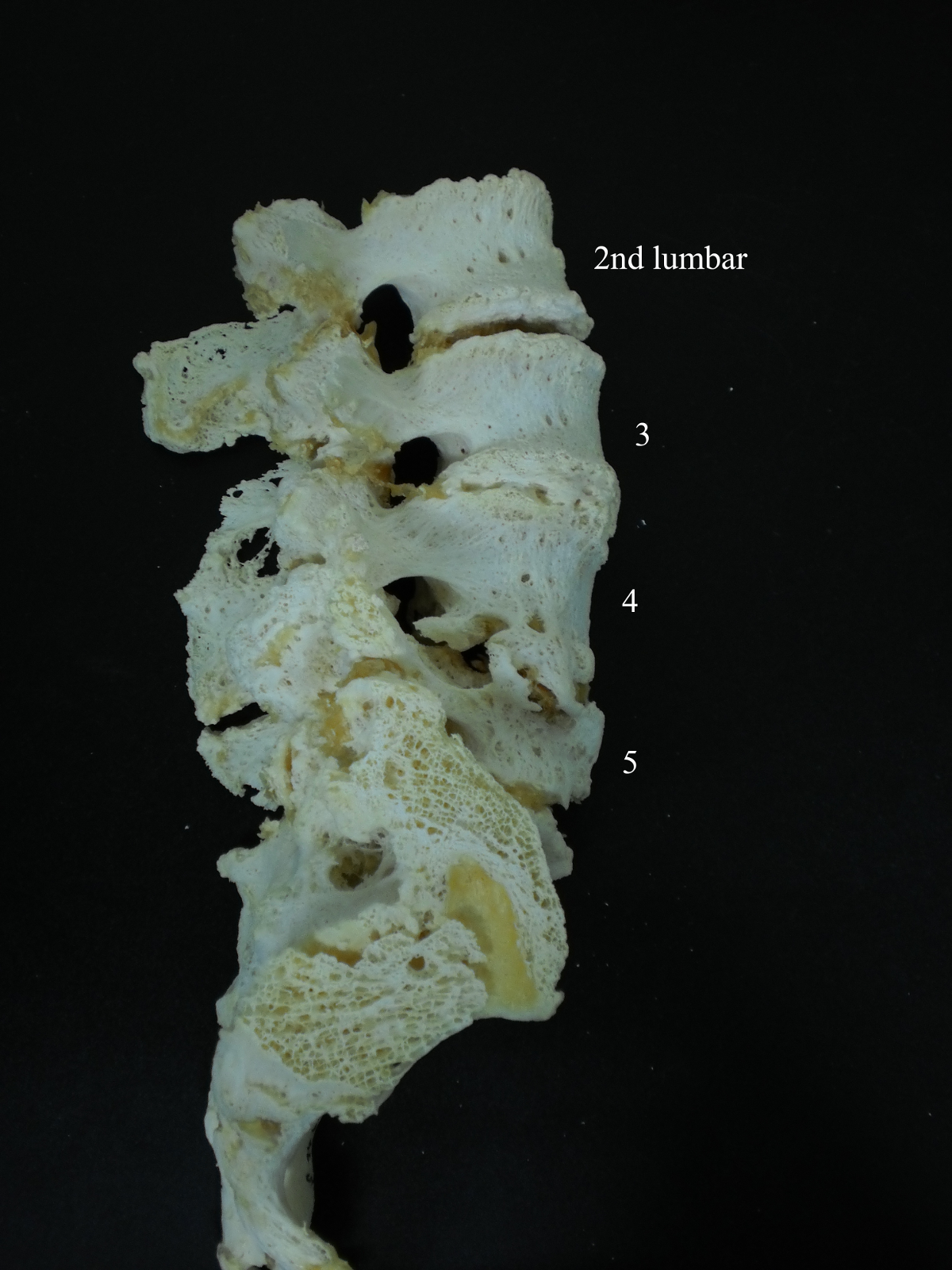

Supplement: Figure S10 — Autopsy Number: 1466, Autopsy Year: 1966, Age: 85, Sex: Female. (TIF) [file pone.0062798.s010.tif]

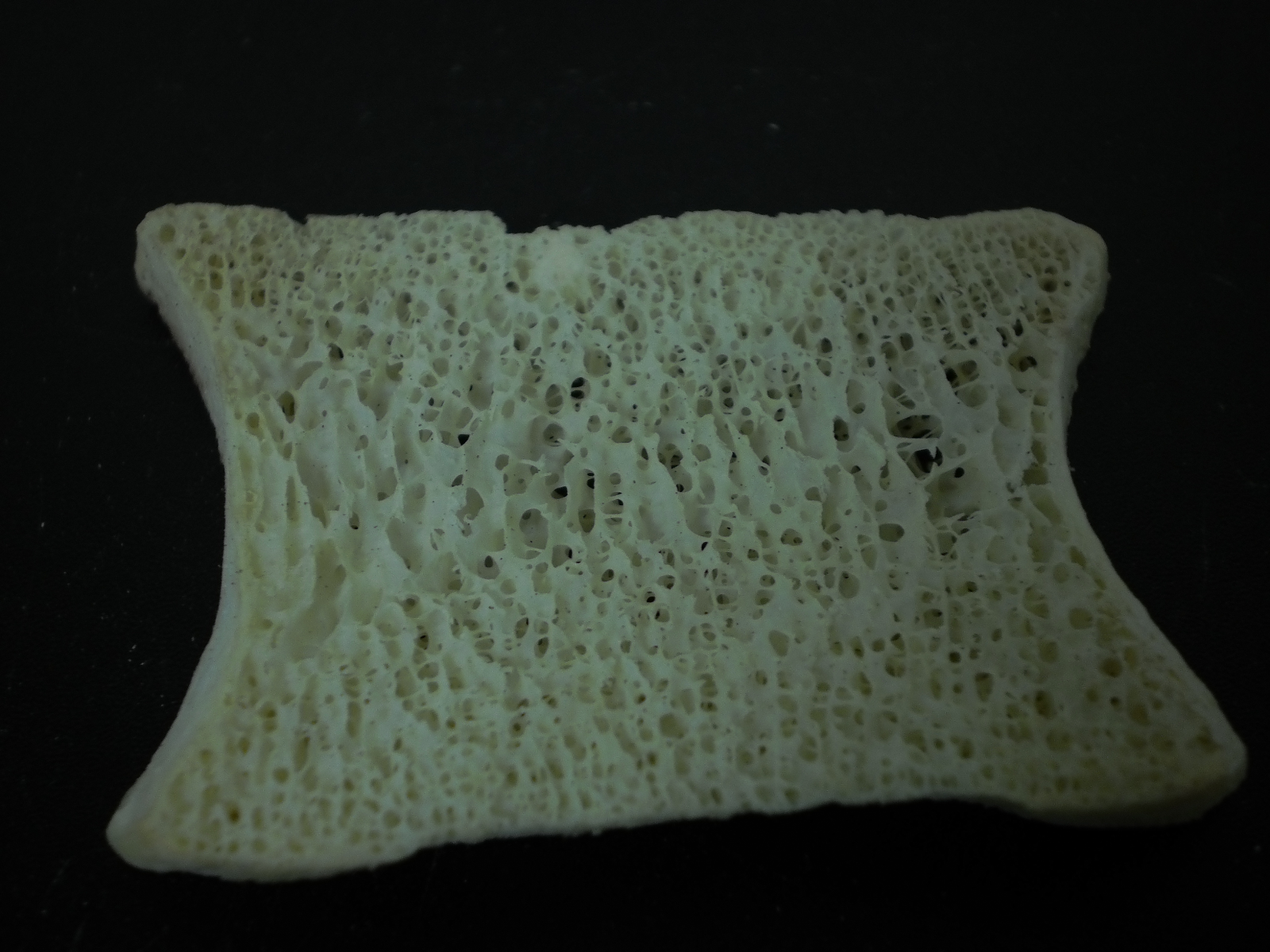

Supplement: Figure S11 — Autopsy Number: 1485, Autopsy Year: 1960, Age: 31, Sex: Female. (TIF) [file pone.0062798.s011.tif]

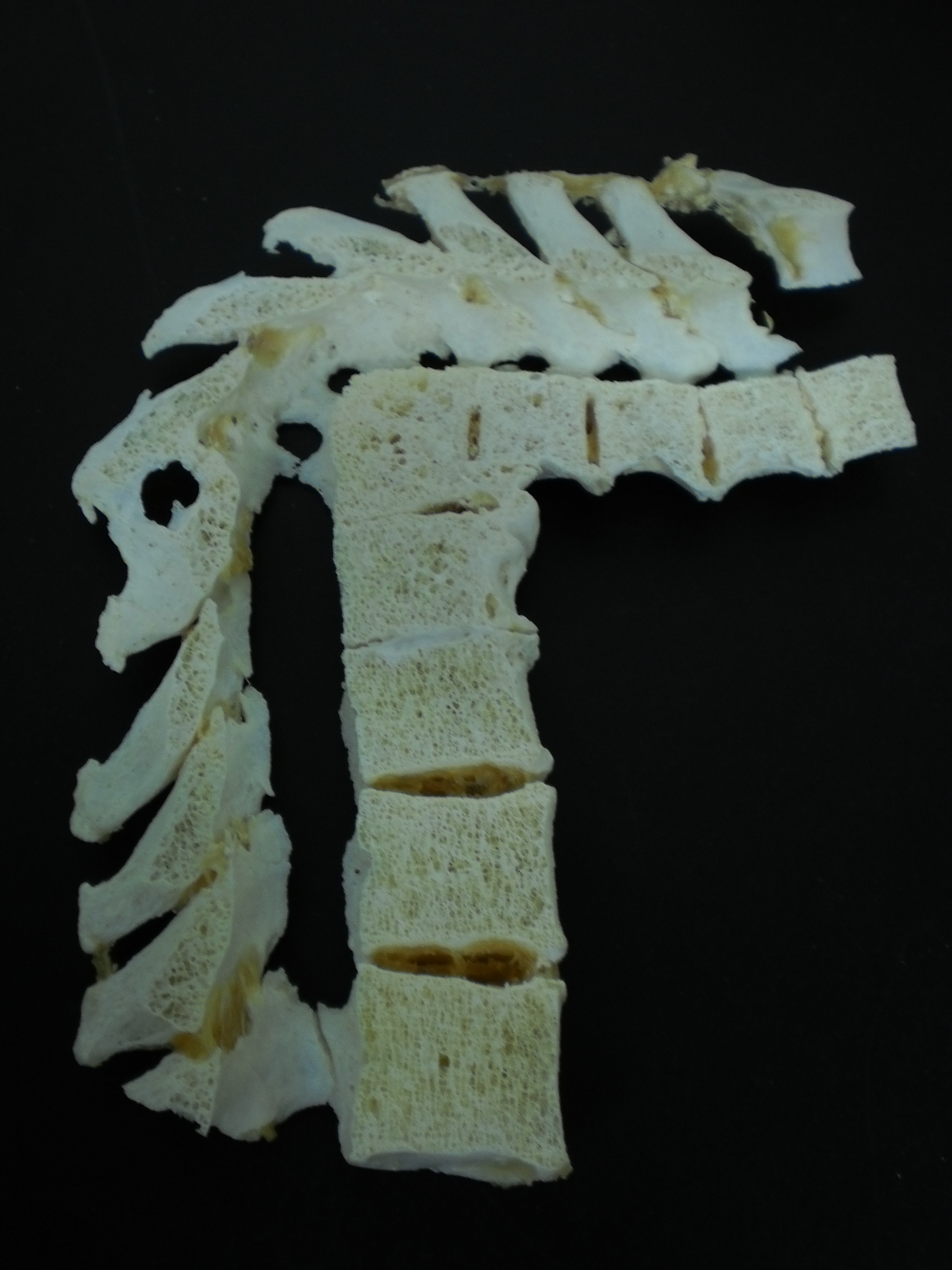

Supplement: Figure S12 — Autopsy Number: 1959, Autopsy Year: 1966, Age: 76, Sex: Female. (TIF) [file pone.0062798.s012.tif]

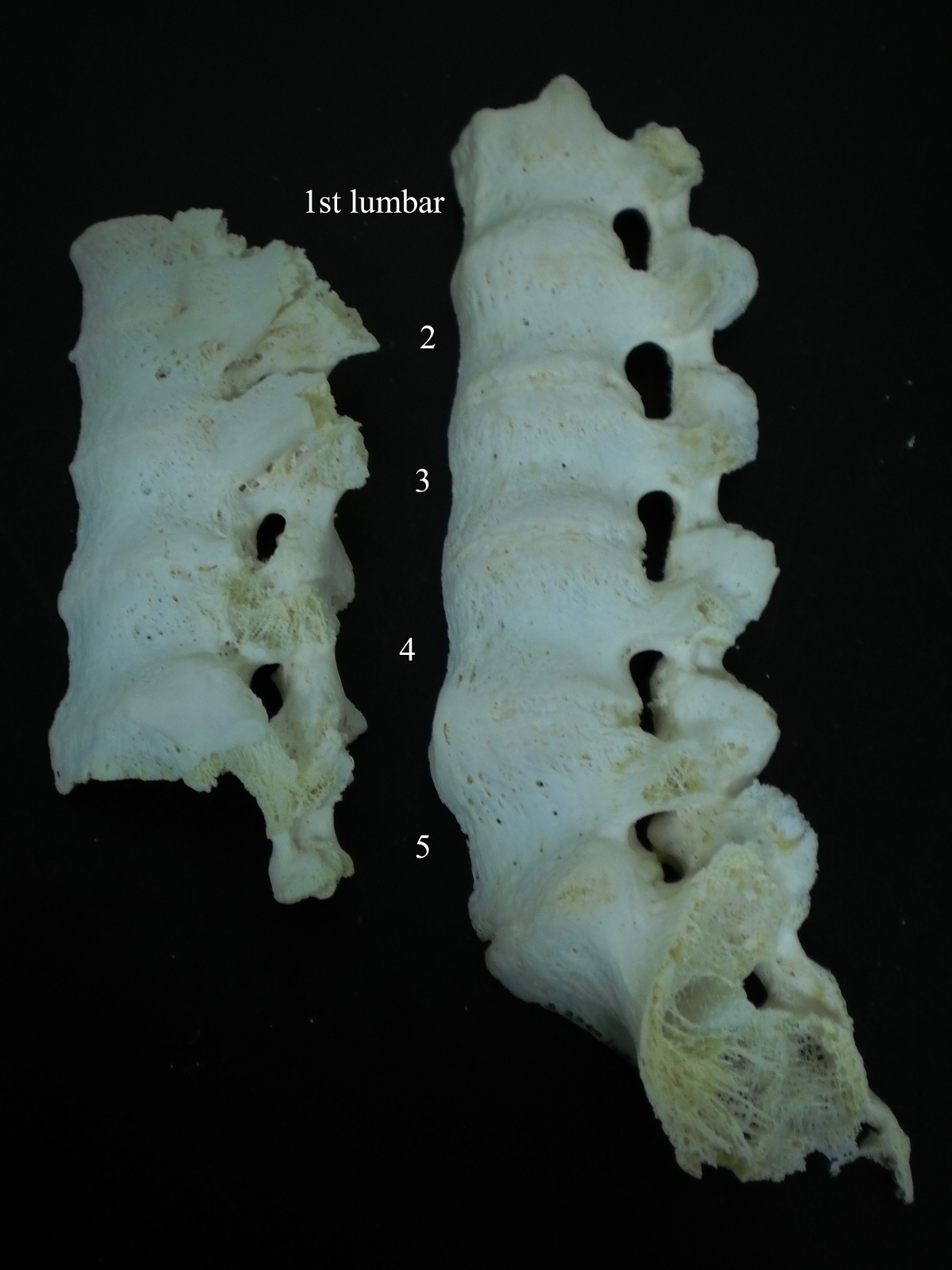

Supplement: Figure S13 — Autopsy Number: 2289, Autopsy Year: 1968, Age: 60, Sex: Male. (TIF) [file pone.0062798.s013.tif]

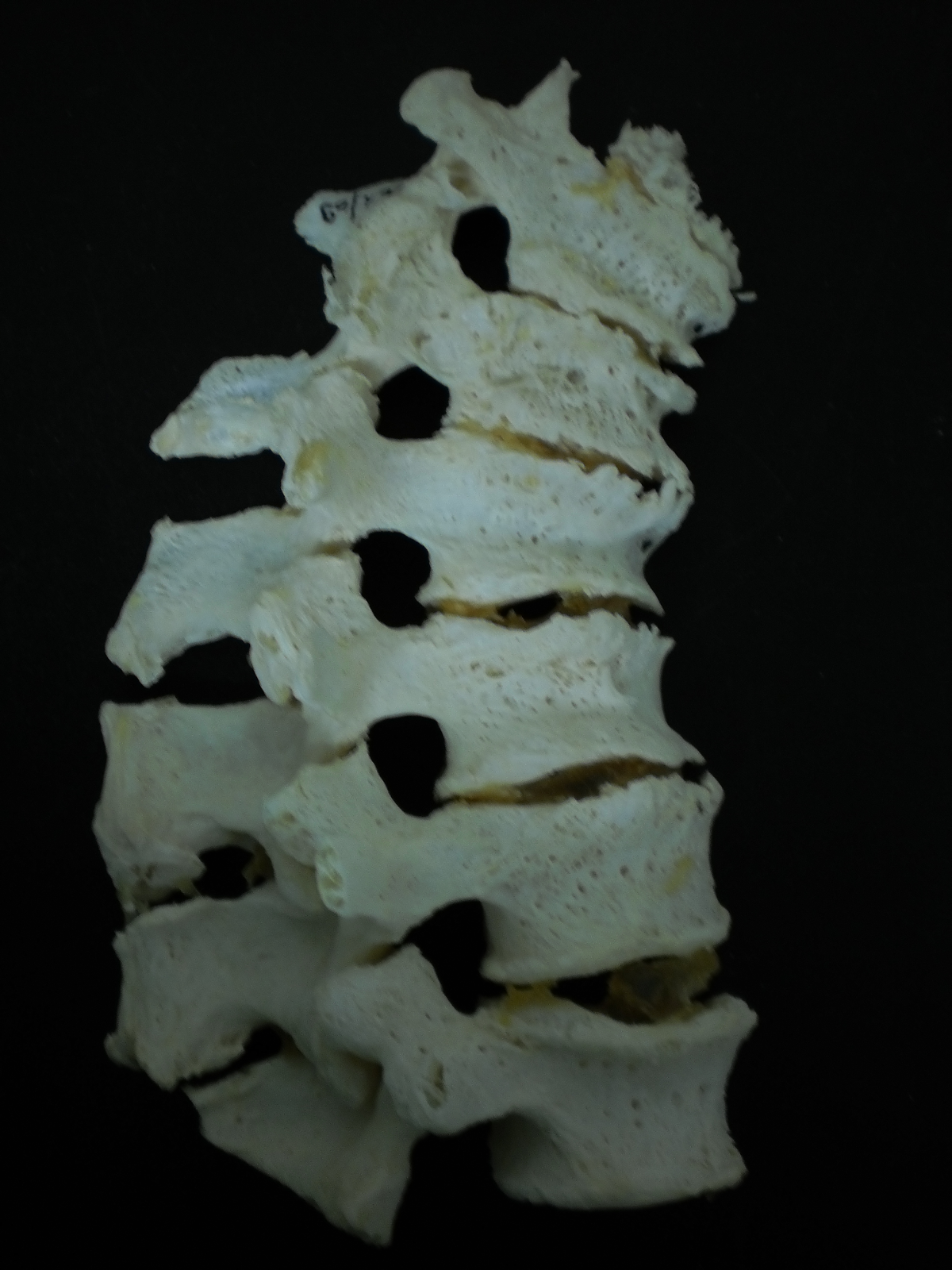

Supplement: Figure S14 — Autopsy Number: 2461, Autopsy Year: 1969, Age: 69, Sex: Female. Note that Figure 1C also shows another image of this case. (TIF) [file pone.0062798.s014.tif]
