# Supplementary material for: Skeletal Lesions in Human Tuberculosis May Sometimes Heal: An Aid to Palaeopathological Diagnoses
Source: PLoS One. 2013 Apr 24;8(4):e62798. doi: 10.1371/journal.pone.0062798 (PMC3634763; doi:10.1371/journal.pone.0062798)
Supplement: Appendix S1 — (PDF) [file pone.0062798.s015.pdf]

Appendix: Additional cases of tuberculosis diagnosed in Galler Collection patients where skeletal lesions have developed. The cases are organised into first (before 1946), second (1946-1950) and third (after 1950) time periods.

<u>Second time period (1946-1950)</u>

Autopsy Number: 739

Autopsy Year: 1948

Age: 76

Sex: Female

Description: A single lumbar vertebra of a 76 year old female was available for study and shows a lytic lesion in the anterior region of the vertebral body. The size of this lesion is approximately 7 mm x 9 mm. No healing has occurred in this case.

Since this individual only had one vertebra available for study, this complicates any potential differential diagnosis. The medical records include information regarding the presence of TB spondylitis as well as an aortic aneurysm. There is a description of vertebral erosion due to the aortic aneurism rather than through TB. However, the spondylitis of TB may have been a confounding factor in the destruction of this vertebra. The bone destruction is on the anterior region of the vertebra, as would be expected in TB. We could not provide a clear diagnosis for this case, but it is possible TB was at least partially responsible for the lesion observed.

<u>Third time period (after 1950)</u>

Autopsy Number: 411

Autopsy Year: 1955

Age: 34

Sex: Male

Description: The image shows the section extending from cervical vertebra six to thoracic vertebra seven of a 34 year old male. Spondylitis of thoracic vertebrae one to three has resulted in extensive fusion along the surface of the vertebral bodies. Intervertebral discs spaces are no longer present. This individual had pulmonary TB in the right lung as the primary focus and the lymph node in the neck was the secondary focus (starting in 1922, 33 years before death). The lymph nodes were later removed surgically. The individual also had meningeal TB (of the inner ear). The meningeal and spinal TB began around 1 year of age. At age 3, this individual was hospitalized and placed in a plaster corset. At the age of 19, the

urogenital system had been affected by TB. As a result of this, the right kidney was removed. During the next 20 years, the individual had several operations to help with the problems caused by urogenital TB. Healing in this case has occurred through fusion of the affected vertebrae.

The medical records for this patient do not describe compression fractures, Paget’s disease, osteomyelitis or neoplasms. Osteoporosis is mentioned, but this would not have impacted the development of the bone lesions because it is very unlikely this individual had osteoporosis at the age of 1 year, when the spinal TB begun. The anterior regions of the bodies of three vertebrae were destroyed, leading to collapse of the spine. This is consistent with TB and we considered this as a very likely case.

SEE FIGURE 1B FOR IMAGE

Autopsy Number: 60

Autopsy Year: 1956

Age: 69

Sex: Male

Description: The image shows the proximal third of both femora, the distal third of the left femur and the second to fifth lumbar vertebrae as well as two sacral vertebrae of a 69 year old male. The fourth and fifth lumbar vertebrae have fused together through their vertebral bodies. There is no intervertebral disc between these vertebrae. The sacral vertebrae were also involved in the disease process. This individual had general subacute miliary TB, as well as meningitis of tuberculous origin. The left femur was also involved as well as the wing of the ilium. Both were atrophied. The left hip was diagnosed with TB in 1954 (2 years before death) when the individual complained of pain in the hip joint and lower extremities. The femoral head had necrotic areas that extended down into the medial part of the compact bone. The joint cartilage had been completely eroded away. Healing occurred through fusion of the affected vertebrae in this case.

The medical record of this individual does mention the presence of Paget’s disease of the femur and third lumbar vertebra. However, pathological changes are noted on lumbar vertebrae four and five, rather than lumbar three. Additionally, the medical records report the presence of tubercles in tissues surrounding lumbar vertebra four, extending further towards the sacrum. There is also complete destruction of the vertebral disc between lumbar vertebrae

four and five. These characteristics indicate that TB was likely responsible for the pathology around lumbar vertebrae four and five.

Autopsy Number: 487

Autopsy Year: 1958

Age: 80

Sex: Male

Description: The image shows the section including thoracic vertebrae eleven to lumbar vertebra five (and the sacrum) of an 80 year old male. The anterior regions of lumbar vertebrae three to five have been almost completely destroyed. Lumbar vertebra two has also been involved and is fused to lumbar vertebra 3 on the anterior edge of the vertebral body. Vertebral bodies are no longer distinct from one another. This individual had pulmonary TB, resulting in spinal lesions. As well as extensive vertebral fusion, healing has occurred by fusion of spinous processes.

The medical records of this individual do not mention compression fractures, Paget’s disease, osteomyelitis or neoplasms but does include hip luxation. However, the lesions we were interested in were on the spine, not the hip. The records reported TB as well as osteoporosis. There is destruction of the anterior part of the vertebral body of lumbar vertebrae three to five with consequent collapse of the spine. Although posterior elements have been involved, there are no indications of other diseases that would have caused this type of lesion.

Autopsy Number: 1441

Autopsy Year: 1954

Age: 80

Sex: Male

Description: The image shows several vertebrae of an 80 year old male, but we were unable to determine specifically which ones these were. Some of the vertebrae have been compressed. Healing has occurred between two vertebrae via fusion of their vertebral bodies. There is also a minor amount of lipping on the vertebra anterior to these two fused vertebrae. The bony deposit protrudes approximately 6 mm from the vertebral body. The fusion is complete leaving no space between vertebrae.

The medical records for this individual state that he had TB spondylitis but not any of the other diseases we considered in our differential diagnoses (compression fractures, Paget’s disease, osteomyelitis or neoplasms). We had a limited number of bones available for

examination; however we do notice the involvement of only two vertebrae. Fusion has occurred between the vertebral bodies of these. Based on the vertebrae, there would have been a mild degree of kyphosis. Based on medical records and these limited observations, we can suggest a possible cause of the vertebral fusion as TB, but we cannot be certain.

Autopsy Number: 2420
Autopsy Year: 1965
Age: 37
Sex: Male
Description: The image shows a view of the right side of the thorax and pelvis of a 37 year old male. The individual had TB spondylitis from age 7, resulting in kyphosis and fusion of thoracic vertebrae nine and ten. The angle of collapse is approximately 90 degrees. Despite the spinal collapse, there is minimal scoliosis. However, the ribs have deformed in order to compensate for the change in the vertebral column. There is also compensation in the lumbar vertebrae. This individual developed measles at the age of 7, followed by pneumonia in both lungs, ultimately resulting in the development of TB and spondylitis. He spent a great amount of time in sanatoria as a child and had to wear a steel corset to keep his chest straight. Healing in this case has occurred by stabilisation of the spine through fusion of thoracic vertebrae.
Medical records for this individual describe severe TB spondylitis, with resulting kyphosis of the spine at an angle of almost 90 degrees, typical of TB. The individual was treated for TB throughout his life, so we were certain that the spinal lesions in this case were the result of TB and not another cause (such as compression fractures, Paget’s disease, osteomyelitis or neoplasms).

Autopsy Number: 1219
Autopsy Year: 1969
Age: 69
Sex: Female
Description: The image shows the right side of the thorax and right humerus of a 69 year old female. In 1918 (51 years before death), this individual had an inflammation of the vertebrae (TB spondylitis), resulting in collapse and fusion of the fifth and sixth thoracic vertebrae. This consequently caused severe kyphoscoliosis in the thoracic and upper lumbar regions of the spine and compensation in the cervical and remaining lumbar regions. The kyphosis/scoliosis involved seven of the thoracic and three of the lumbar vertebrae. The ribs

have been deformed as a result of the spinal collapse. This individual had extensive pulmonary TB. Healing of the spinal lesions in this case has occurred through fusion of the affected vertebrae.

Medical records do not mention any of the other diseases we considered to be the most likely cause of spinal lesions in this sample (compression fractures, Paget's disease, osteomyelitis or neoplasms). The lesions we observed are consistent with TB being the cause. There was anterior destruction of vertebral bodies, leading to a collapse of the spine in the thoracic region. Only two vertebrae were affected by the disease process. Posterior regions of the vertebrae were unaffected. In this case, the lesions of the spine would be caused by TB spondylitis.

Autopsy Number: 1645

Autopsy Year: 1957

Age: 43

Sex: Female

Description: The image shows a single lumbar vertebra of a 43 year old female. We were unable to determine specifically which vertebra. A circular lytic lesion is present on the right side of the vertebral body measuring approximately 12 mm x 14 mm. This individual had pulmonary TB of the left lung. In this case, there is no evidence of healing.

The medical records for this individual do not mention any of the other diseases we considered (compression fractures, Paget's disease, osteomyelitis or neoplasms). There is mention of TB spondylitis, however. In this case, we only had a single lumbar vertebra available for examination. Destruction of the central part of the vertebral body is not uncommon in TB and the destruction affected only a single lumbar vertebra. We could not tell anything about potential kyphosis for this case; however, with the bone destruction observed, it would be unlikely that the spine would have collapsed. This individual died at an earlier than average age of 43 years (average was 62±2 years) of a haemorrhage in the brain. It is possible that this individual died before bone lesions resulting from TB could develop further.

SEE FIGURE 1A FOR IMAGE

Autopsy Number: 785

Autopsy Year: 1963

Age: 64

Sex: Male

Description: The first image shows the seventh to tenth thoracic vertebrae and the second shows three higher thoracic vertebrae of a 64 year old male. The individual had pulmonary TB as well as TB spondylitis of the eighth thoracic vertebra, which also later affected the ninth and tenth thoracic vertebrae. The anterior region of thoracic vertebra nine has been almost completely destroyed. The eighth and tenth thoracic vertebrae have fused to the ninth through the surface of their vertebral bodies. Thoracic vertebra seven appears to have two lytic lesions on the anterior region of the vertebral body. The first (positioned superiorly) measures approximately 1.5 mm x 2.5 mm. The second measures 3.5 mm x 2.5 mm.

There is also bone deposition and lipping on the other lumbar vertebrae (though these are not fused together). The vertebra in the centre of the second image has a small, circular lytic lesion on the left side of the vertebral body. It measures approximately 2.2 mm x 1.8 mm. Thoracic vertebrae eight to ten have healed through fusion of the vertebrae as well as posterior elements.

The medical records for this individual describe TB spondylitis of multiple vertebrae in detail as well as the presence of osteoporosis. There were two separate foci for skeletal lesions; the first of four thoracic vertebrae and the second involved two lumbar vertebrae. Although a total of six vertebrae were affected, atypical for TB, these vertebrae were not adjacent to one another, making TB still a potential cause of the lesions. Additionally, for the thoracic vertebrae, there was extensive destruction of the anterior region of the vertebral bodies. One vertebra has been almost completely destroyed. This has led to collapse of the spine and fusion of the affected vertebrae. Although posterior elements were involved, the most probable cause of these lesions is likely to be TB.

SEE FIGURE 1D FOR FIRST HALF OF THIS IMAGE

Autopsy Number: 1167

Autopsy Year: 1960

Age: 94

Sex: Female

Description: The image shows a lumbar vertebra as well as four other vertebrae (possibly thoracic) of a 94 year old female. The individual had bone TB causing some damage to lower

thoracic and upper lumbar vertebrae. The medical records also describe kyphoscoliosis of the second to fourth lumbar vertebrae. In this case, healing has occurred via bone remodelling and deposition.

Medical records for this individual report bone TB, osteoporosis and kyphoscoliosis of lumbar vertebrae two to four. There is no mention of compression fractures, Paget’s disease, osteomyelitis or neoplasms. It may be that the visible vertebral malformations are a result of aging. We had limited skeletal material available for examination in this case, which excluded lumbar vertebrae two to four. Based on the material we did have available, we observed some damage to the vertebrae and no evidence of other diseases. In this case, we had to base most of the diagnosis from the medical records, but considered this individual to have TB as described in the reports.

Autopsy Number: 1227

Autopsy Year: 1969

Age: 89

Sex: Female

Description: The image shows three thoracic vertebrae of an 89 year old female. We were unable to determine which vertebrae these were specifically; however, none of them were the fifth thoracic vertebra. There was fusion and bone deposition around several costovertebral joints as well as small amounts of bone deposition between vertebrae. The anterior region of two thoracic vertebrae (lower two shown in the image) have fused together, however, this fusion is not complete between vertebral bodies. The medical records also describe TB spondylitis of the fifth thoracic vertebra, with small abscesses and kyphosis. The vertebra is almost completely destroyed. Healing in this case is a result of bone deposition.

Medical records for this individual describe TB spondylitis of the fifth thoracic vertebra as well as osteoporosis, but not compression fractures, Paget’s disease, osteomyelitis or neoplasms. The fifth thoracic vertebra was not available for examination; the medical reports indicated that it had been almost completely destroyed by the disease process. Kyphosis resulted from this event. Observations from the vertebrae available revealed evidence of vertebral fusion. Due to the limited number of vertebrae involved, reports of vertebral destruction and spinal collapse, this can be considered a case of TB.

Autopsy Number: 1466

Autopsy Year: 1966

Age: 85

Sex: Female

Description: The image shows the second to fifth lumbar vertebrae and the sacrum of an 85 year old female. The individual had TB spondylitis resulting in destruction of the anterior region of the fifth lumbar vertebra. The vertebral body is almost completely destroyed. Lipping and bone deposition has occurred on several vertebrae. This included the inferior edge of the vertebral body of the third and fourth lumbar vertebrae. Some fusion of posterior elements of lumbar vertebrae three to five have occurred.

This individual was reported to have spondylitis TB, osteoporosis, kyphosis and gonarthrosis. The lower vertebrae were available for examination and anterior destruction of a single vertebral body was observed. Since this was the fifth lumbar vertebra, collapse of the spine was very minimal. Lytic lesions are present on the fourth lumbar vertebra. There was a large amount of bone deposition in this case; however, the individual was 85 years at the time of death. Thus these lesions could be the result of many years of healing after the disease was arrested.

Autopsy Number: 1485

Autopsy Year: 1960

Age: 31

Sex: Female

Description: The image shows a single vertebra of a 31 year old female, though we were unable to determine the specific vertebra. It is slightly compressed but this may not be related to a tuberculosis disease process. This patient had chronic TB that also showed evidence of involvement of the psoas muscle, which had healed by bone deposition on the femur.

Medical records for this individual describe chronic TB as well as several cardiac conditions. This individual may not have developed spinal TB as she died at 31 years, much earlier than the average for the sample (62±2 years). In this case, we considered the individual to have TB of the hip joint and psoas muscle, but not of the spine.

Autopsy Number: 1959

Autopsy Year: 1966

Age: 76

Sex: Female

Description: The image shows twelve vertebrae (probably all thoracic) of a 76 year old female. This individual had pulmonary TB for many years. Five of the middle thoracic vertebrae have been destroyed in the anterior region of the vertebral body. This had led to collapse of the spine at a 90 degree angle. These five thoracic vertebrae have fused together into a solid mass, making it impossible to distinguish single vertebrae. Posterior elements near to the site of collapse have fused together, potentially as a means of mechanical stabilisation for the spine.

Medical records for this individual give details of both breast and lung cancers, which were surgically removed. “Old” pulmonary TB as well as a description of the spinal lesions observed were also given. Although it is possible that the metastases were responsible for the lesions observed, this is unlikely. There is specific destruction of the anterior regions of the vertebral bodies, with marked kyphosis (angle approximately 90 degrees) and fusion of the affected vertebrae. Five vertebrae were affected, however, this individual had TB for many years and bone destruction is extensive. Posterior elements are involved, however this would have occurred some time after the disease was arrested. Differential diagnosis in this case indicates the lesions were very likely to be a result of TB.

Autopsy Number: 2289

Autopsy Year: 1968

Age: 60

Sex: Male

Description: The image shows four thoracic vertebrae (left) and lumbar vertebrae one to five plus the upper part of the sacrum (right) of a 60 year old male. This is the only individual with the cause of death recorded as tuberculosis. He had pulmonary TB in the right lung and received a lobectomy of the upper lobe 3 years before death. There are lytic lesions on vertebral bodies. The individual also had ankylosing spondylitis which would have strengthened the spine after bone destruction from a tuberculosis related process.

This individual suffered from a compression fracture of thoracic vertebra twelve, although it could have also been a result of a metastatic growth. Ankylosing spondylitis as well as pulmonary TB, the latter of which, was the cause of death. In this case, it was difficult to

determine the cause of any observed skeletal lesions because the ankylosing spondylitis covered any lytic lesions that may have been present.

Autopsy Number: 2461

Autopsy Year: 1969

Age: 69

Sex: Female

Description: The images show six vertebrae (left) and five vertebrae (right), most likely thoracic and lumbar, of a 69 year old female. The individual had chronic TB and spondylitis. The cause of death was pneumonia and may have been the result of complications arising from TB. Lipping and bone deposition has occurred on several vertebrae. Between two vertebrae (right) enough bone has been deposited to fuse the two together at the anterior edge of the vertebral body. There is also bone deposition on several ribs. One costovertebral joint is fused.

Medical records for this individual reported osteoporosis, kyphosis, spondylosis and chronic TB, but not compression fractures, Paget’s disease, osteomyelitis or neoplasms. Observations from the vertebrae show damage to vertebral bodies as well as bone deposition. There is a mild kyphosis (as observed in the left image). In the absence of any other evidence, it was difficult to diagnose this case. The most likely cause of these lesions is a combination of TB and osteoporosis.

SEE FIGURE 1C FOR SECOND PART OF THIS IMAGE
